# GhCASPL1 regulates secondary cell wall thickening in cotton fibers by stabilizing the cellulose synthase complex on the plasma membrane

**DOI:** 10.1111/jipb.13777

**Published:** 2024-09-24

**Authors:** Li Zhang, Xingpeng Wen, Xin Chen, Yifan Zhou, Kun Wang, Yuxian Zhu

**Affiliations:** ^1^ Institute for Advanced Studies Wuhan University Wuhan 430072 China; ^2^ State Key Laboratory of Hybrid Rice, Hubei Hongshan Laboratory, College of Life Sciences Wuhan University Wuhan 430072 China; ^3^ TaiKang Center for Life and Medical Sciences Wuhan University Wuhan 430072 China; ^4^ State Key Laboratory of Protein and Plant Gene Research, College of Life Sciences Peking University Beijing 100871 China

**Keywords:** cellulose synthase complex (CSC), cotton fiber, GhCASPL1, plasma membrane (PM), secondary cell wall (SCW)

## Abstract

Cotton (*Gossypium hirsutum*) fibers are elongated single cells that rapidly accumulate cellulose during secondary cell wall (SCW) thickening, which requires cellulose synthase complex (CSC) activity. Here, we describe the CSC‐interacting factor CASPARIAN STRIP MEMBRANE DOMAIN‐LIKE1 (GhCASPL1), which contributes to SCW thickening by influencing CSC stability on the plasma membrane. *GhCASPL1* is preferentially expressed in fiber cells during SCW biosynthesis and encodes a MARVEL domain protein. The *ghcaspl1 ghcaspl2* mutant exhibited reduced plant height and produced mature fibers with fewer natural twists, lower tensile strength, and a thinner SCW compared to the wild type. Similarly, the Arabidopsis (*Arabidopsis thaliana*) *caspl1 caspl2* double mutant showed a lower cellulose content and thinner cell walls in the stem vasculature than the wild type but normal plant morphology. Introducing the cotton gene *GhCASPL1* successfully restored the reduced cellulose content of the Arabidopsis *caspl1 caspl2* mutant. Detergent treatments, ultracentrifugation assays, and enzymatic assays showed that the CSC in the *ghcaspl1 ghcaspl2* double mutant showed reduced membrane binding and decreased enzyme activity compared to the wild type. GhCASPL1 binds strongly to phosphatidic acid (PA), which is present in much higher amounts in thickening fiber cells compared to ovules and leaves. Mutating the PA‐binding site in GhCASPL1 resulted in the loss of its colocalization with GhCesA8, and it failed to localize to the plasma membrane. PA may alter membrane structure to facilitate protein–protein interactions, suggesting that GhCASPL1 and PA collaboratively stabilize the CSC. Our findings shed light on CASPL functions and the molecular machinery behind SCW biosynthesis in cotton fibers.

## Graphical abstract

The cellulose synthase complex–interacting factor CASPARIAN STRIP MEMBRANE DOMAIN‐LIKE1 plays a crucial role in regulating secondary cell wall thickening in cotton fibers by stabilizing the cellulose synthase complex on the plasma membrane.

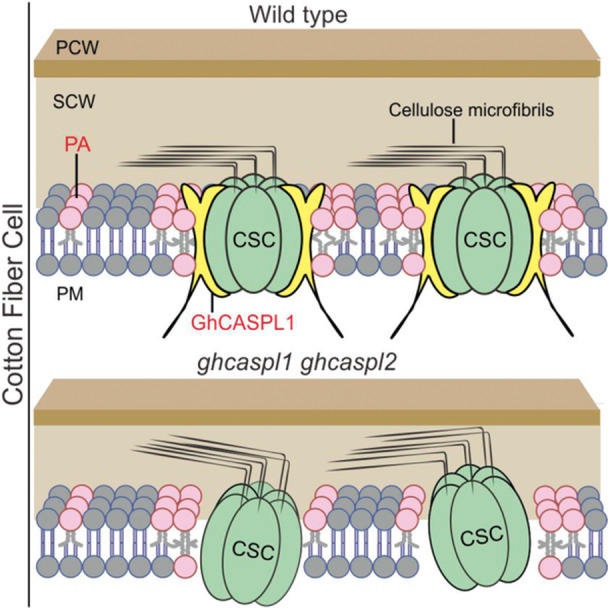

## INTRODUCTION

Cellulose, the most abundant biopolymer on Earth, is synthesized on the plasma membrane (PM) and crystallizes into microfibrils, which are the main load‐bearing components of cell walls (Li et al., 2021; Zhang et al., 2021; Wolf, 2022). Cotton fiber, composed of over 95% cellulose, is harvested from the cotton plant (*Gossypium hirsutum*), the largest fiber crop globally and the most common natural material used by the textile industry (Qin and Zhu, 2011; Huang et al., 2021; Wen et al., 2023). Cotton fiber not only plays a significant role in the global economy but also serves as an ideal material for studying cellulose biosynthesis (Haigler et al., 2012). Mature cotton fiber cells mainly consist of cellulose microfibrils that are synthesized during secondary cell wall (SCW) deposition via concerted enzymatic activity catalyzed by the cellulose synthase complex (CSC), composed of equimolar amounts of the *G. hirsutum* cellulose synthases GhCesA4, GhCesA7, and GhCesA8 (Wen et al., 2022).

Secondary cell wall formation is characterized by a significantly higher rate of CSC transport compared to primary cell wall (PCW) formation, which is indicative of greater cellulose biosynthesis during SCW development (Wightman et al., 2009; Watanabe et al., 2015). Scientists have been investigating the mechanisms underlying the regulation of cellulose synthase in the PCW of Arabidopsis, identifying several key regulatory proteins (Polko and Kieber, 2019; Purushotham et al., 2020). Notably, the cellulose synthase interactive 1 (CSI1) protein interacts with the CSC and directly binds to microtubules, facilitating the movement of CSC along the microtubule direction (Bringmann et al., 2012; Li et al., 2012). Furthermore, CSI1 works in conjunction with the EXOCYST complex and secretory vesicles to transport CSCs during the development of the PCW, which plays a crucial role in determining the rate of CSC transport to the PM (Zhu et al., 2018). But, capturing the specific SCW regulatory factors that modulate CSC activity is challenging as SCWs are usually limited to vascular bundles of the root and stem in most plants. To date, only the β‐1,4‐glucanase KORRIGAN, the putative glycosylphosphatidylinositol (GPI)‐anchored extracellular protein COBRA, and the Golgi‐localized proteins STELLO1/2 have been shown to interact with CSCs to regulate SCW formation (Roudier et al., 2002; Mansoori et al., 2014; Vain et al., 2014; Zhang et al., 2016).

Many specialized cell wall regions serve as diffusion barriers for the development of various secondary tissues in plants (Geldner, 2013). One such well‐known barrier is the Casparian strip, which shares similarities with SCWs. CASPARIAN STRIP MEMBRANE DOMAIN PROTEINs (CASPs) localize to the Casparian strip domain during its formation (Roppolo et al., 2011; Roppolo et al., 2014). The small CASPARIAN STRIP MEMBRANE DOMAIN‐LIKE (CASPL) proteins are also known as MARVEL protein family members in non‐plant systems (Roppolo et al., 2014). The MARVEL (MAL and related proteins for vesicle trafficking and membrane link) domain is characterized by a four transmembrane‐helix architecture (Sanchez‐Pulido et al., 2002). Proteins containing the MARVEL domain play crucial roles in both physiological and pathological processes in humans, particularly in cholesterol‐rich membrane apposition events involved in various cellular processes such as vesicle formation and tight junction assembly (Magal et al., 2009; Yaffe et al., 2012). Most CASPLs were able to localize to the Casparian strip domain when their encoding genes were ectopically expressed in the endodermis (Roppolo et al., 2014). However, the function of the CASPL family remains unknown. *CASPL*s in Arabidopsis (*Arabidopsis thaliana*) were shown to be specifically expressed in trichomes, peripheral root cap cells, xylem pericycle cells, and abscission zone cells (Roppolo et al., 2014; Champeyroux et al., 2019). Most of these cells show polar or local SCW modifications. For instance, endothecium cell walls in developing anthers undergo lateral thickening, leading to the formation of bar‐like structures rich in cellulose and lignin (Dawson et al., 1999). CASPLs are thought to have specialized functions similar to CASPs in forming a membrane scaffold at the Casparian strip and/or recruiting cell wall‐modifying enzymes (Roppolo et al., 2011; Roppolo et al., 2014).

In this study, we showed that GhCASPL1, a member of the CASPL family from cotton, interacts with the CSC and binds to phosphatidic acid (PA) in the cell membrane. This interaction preserves the stability of the CSC on the PM, thereby maintaining its normal cellulose synthesis activity and promoting SCW thickening. Our study provides evidence that CASPL is a membrane scaffold cofactor which enhances cellulose biosynthesis, increasing our understanding of SCW thickening during cell development in cotton fibers.

## RESULTS

### GhCASPL1 interacts with *Gossypium hirsutum* cellulose synthase complex (CSC) components

**Figure 1:**
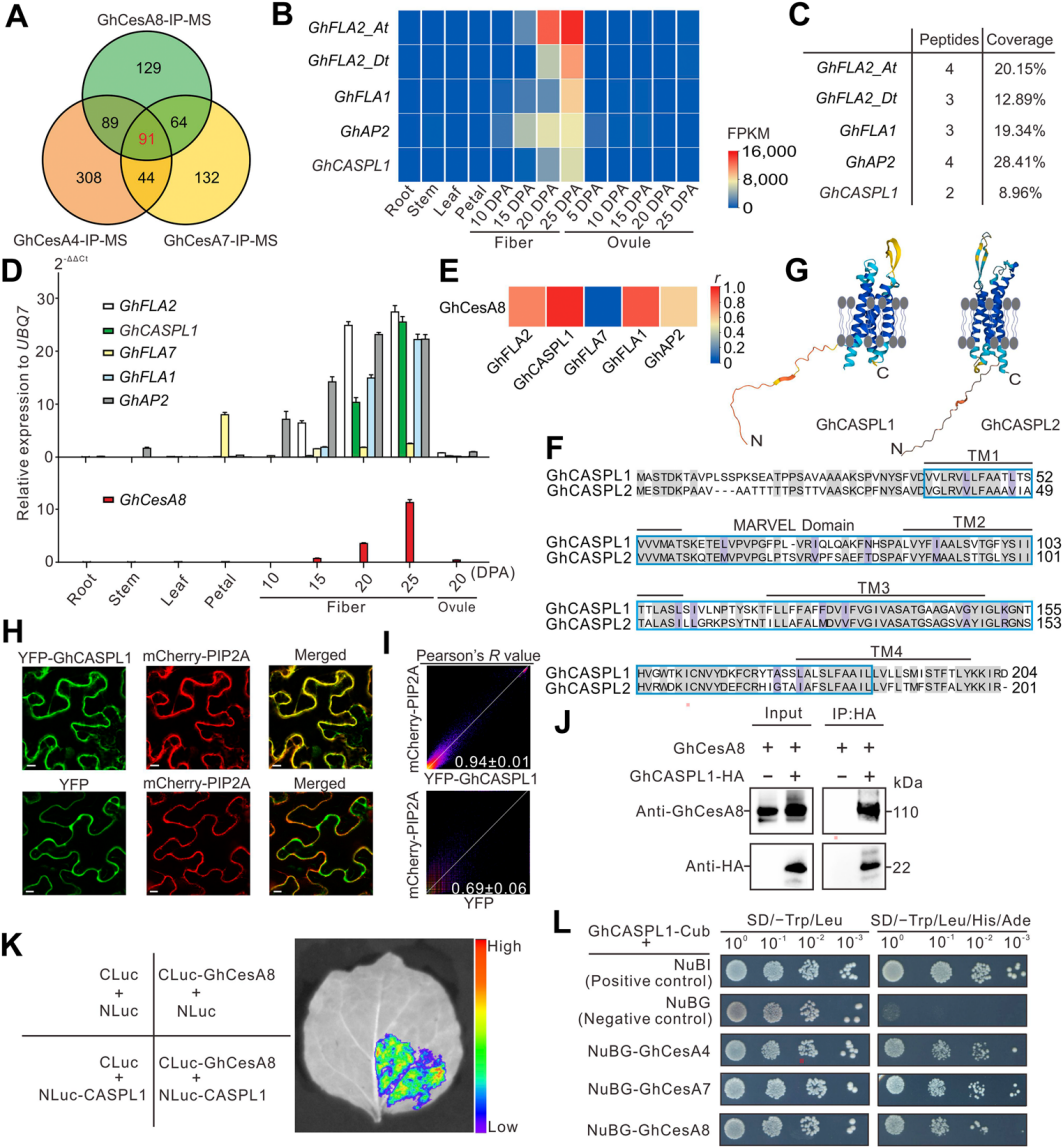
***Gossypium hirsutum*** **CASPARIAN STRIP MEMBRANE DOMAIN‐LIKE1 (GhCASPL1) is a novel cellulose synthase complex (CSC)‐interacting factor that may participate in secondary cell wall (SCW) formation in cotton fibers** **(A)** The overlap between putative CSC‐interacting proteins identified by immunoprecipitation–mass spectrometry (IP–MS) using anti‐*G. hirsutum* cellulose synthase A4 (anti‐GhCesA4), anti‐GhCesA7, and anti‐GhCesA8 antibodies. **(B)** Expression patterns of the genes encoding the top five SCW‐related proteins among 91 common proteins that co‐immunoprecipitated with GhCesA4, GhCesA7, and GhCesA8. **(C)** Summary of peptides and coverage information for the top five immunoprecipitated proteins. Data are based on three independent IP‐MS experiments. **(D)** *GhCASPL1* is predominantly expressed during SCW formation in fiber cells (top) and follows the same pattern as the SCW‐specific gene *GhCesA8* (bottom). *UBQ7* was used as a normalization control. Values are means ± *SD* from three independent biological replicates. **(E)** Correlation analysis between the top five immunoprecipitated proteins and *GhCesA8* expression patterns in various cotton tissues and cells using the RSQ function (calculate the square of the correlation coefficient). **(F)** Alignment of GhCASPL1 and GhCASPL2 protein sequences, showing that they share 63.5% sequence identity. The shared typical MARVEL domain and the four transmembrane (TM1–4) regions are shown. **(G)** Protein structure prediction by AlphaFold showing that GhCASPL1 and GhCASPL2 have a disordered N‐terminal region and that their four transmembrane helices span the entire membrane. **(H)** Confocal microscopy indicating that the yellow fluorescent protein (YFP)‐GhCASPL1 fusion protein co‐localized with mCherry‐PIP2A, a plasma membrane marker, implying that YFP‐GhCASPL1 is indeed located in the plasma membrane. YFP and mCherry‐PIP2A were used as the negative controls. Scale bars, 10 μm. **(I)** Analysis of Pearson's *r* values to assess colocalization of the fusion proteins in H. A strong correlation was observed between YFP‐GhCASPL1 and mCherry‐PIP2A (*r* = 0.94 ± 0.01), while the negative control YFP and mCherry‐PIP2A showed a weaker correlation (*r* = 0.69 ± 0.06). **(J)** Co‐immunoprecipitation (Co‐IP) assay showing that GhCASPL1‐HA (hemagglutinin) interacts with GhCesA8. Total microsomal proteins (input) and the immunoprecipitated fractions (IP:HA) were analyzed by immunoblotting with anti‐HA and anti‐GhCesA8 antibodies. **(K)** Split luciferase assay showing that GhCASPL1 and GhCesA8 interact *in planta*. The constructs *NLuc‐GhCASPL1* and *CLuc‐GhCesA8* were co‐expressed in *Nicotiana benthamiana* leaves, which were imaged for luciferase activity 48 h later. **(L)** Membrane‐based yeast two‐hybrid assay of the interactions between GhCASPL1 and GhCesA4, GhCesA7, and GhCesA8. The N‐terminus of each GhCesA protein was fused to the NubG domain of ubiquitin; the Cub domain was fused to the C‐terminus of GhCASPL1. NubI and NubG were used as positive and negative controls, respectively.

To identify proteins that bind to the major CSC component cellulose synthase, we prepared microsomal fractions from developing cotton fibers (Wen et al., 2022) and subjected them to immunoprecipitation (IP) with specific antibodies against GhCesA4, GhCesA7, or GhCesA8. We identified the co‐precipitating proteins by mass spectrometry (MS), resulting in several hundred potential target proteins from each independent IP, 91 of which were shared by all three IP–MS trials (Figure 1A; Table S1).

To identify interactors of cellulose synthase, we focused on proteins in which genes are highly expressed in SCWs in developing cotton fibers based on published RNA sequencing (RNA‐seq) data. Five genes caught our attention, as they were among the most highly expressed genes in developing fibers: three fasciclin‐like arabinogalactan genes (*GhFLA2*, *GhFLA7*, and *GhFLA1*), one gene encoding an arabinogalactan protein (*GhAP2*), and one member of the *CASPL* family, *GhCASPL1* (Figure 1B; Table S2). Further analysis of our MS data revealed multiple peptides with relatively high sequence coverage for each protein (Figure 1C). Reverse transcription – quantitative polymerase chain reaction (RT‐qPCR) analysis indicated that their encoding genes are predominantly expressed during SCW formation, with the expression pattern of *GhCASPL1* exhibiting the strongest correlation (correlation coefficient *r* = 0.97) with that of the SCW‐specific gene *GhCesA8* (Figure 1D, E) (Wen et al., 2022).

Using the GhCASPL1 protein sequence as a query on the Cottongen website, we identified 12 homologous proteins encoded by the *G. hirsutum* genome (Figures S1, S2a, comprising genes from the At and Dt subgenomes). Most *GhCASPL1*‐related genes were expressed at extremely low levels in the organs or tissues analyzed, except for *GhCASPL1*, *GhCASPL2*, and *GhCASPL3* (Figure S2b). Multiple protein sequence alignment showed that GhCASPL2 shared the highest sequence identity (63.5%) with GhCASPL1; both proteins contain four transmembrane domains with a conserved MARVEL domain (1.65e−38 and 3.94e−33), as determined by a search for conserved domains in National Center for Biotechnology Information Conserved Domain (NCBI CD)‐Search (Figures 1F, S1). MARVEL domain‐containing proteins, such as Occludin and other MAL family members, were reported to have important functions in membrane attachment in animal systems (de Marco et al., 2002; Sanchez‐Pulido et al., 2002). GhCASPL1 and GhCASPL2 were predicted by AlphaFold to take on an M‐shaped topology with four transmembrane helices (Figure 1G). Confocal microscopy revealed colocalization of yellow fluorescent protein (YFP)‐GhCASPL1 and mCherry‐PIP2A (a PM marker), indicating that GhCASPL1 localizes to the PM (Figure 1H). Pearson's correlation analysis indicated a strong correlation (*r* = 0.94 ± 0.01) between YFP‐GhCASPL1 and mCherry‐PIP2A, whereas the negative control, YFP and mCherry‐PIP2A, showed a correlation coefficient of *r* = 0.69 ± 0.06 (Figure 1I). In addition, consistent with the results of GhCASPL1, the homologous protein GhCASPL2 is also localized in the PM (Figure S3).

We attempted to generate specific antibodies against several peptides for GhCASPL1 but failed to obtain a working antibody (data not shown). As an alternative, we generated a transgenic cotton line harboring a transgene consisting of the endogenous *GhCASPL1* promoter driving the expression of *GhCASPL1* cloned in‐frame and upstream of the sequence encoding the hemagglutinin (HA) tag (*proGhCASPL1*:*GhCASPL1‐HA*) (Figure S4). We then performed IP–MS and co‐immunoprecipitation (Co‐IP) assays with an anti‐HA antibody using microsomal fractions obtained from developing cotton fiber cells (Figure S5, upper panel). Among the 69 interacting proteins repeatedly identified across three independent replicates, we detected five peptides from GhCASPL1, representing a protein coverage of 28.9%, and 10 peptides from GhCesA8, representing a coverage of 18.0% (Figure S5), confirming the interaction of GhCSPL1 and GhCesA8. In a Co‐IP assay of the *proGhCASPL1*:*GhCASPL1‐HA* transgenic line using an anti‐HA antibody, GhCesA8 was among the proteins that co‐precipitated with GhCASPL1 (Figure 1J). Split luciferase complementation assays in *Nicotiana benthamiana* leaves confirmed the GhCesA4/7/8–GhCSPL1 interaction (Figures 1K, S6). As GhCSPL1 is predicted to be a membrane protein, we conducted a DUAL‐membrane yeast two‐hybrid assay (Figure 1L), which suggested that GhCASPL1 interacts with GhCesA8, as well as the two other CSC subunits: GhCesA4 and GhCesA7.

### Production of *ghcaspl1 ghcaspl2* homozygous knockout cotton lines and phenotypic analysis

**Figure 2:**
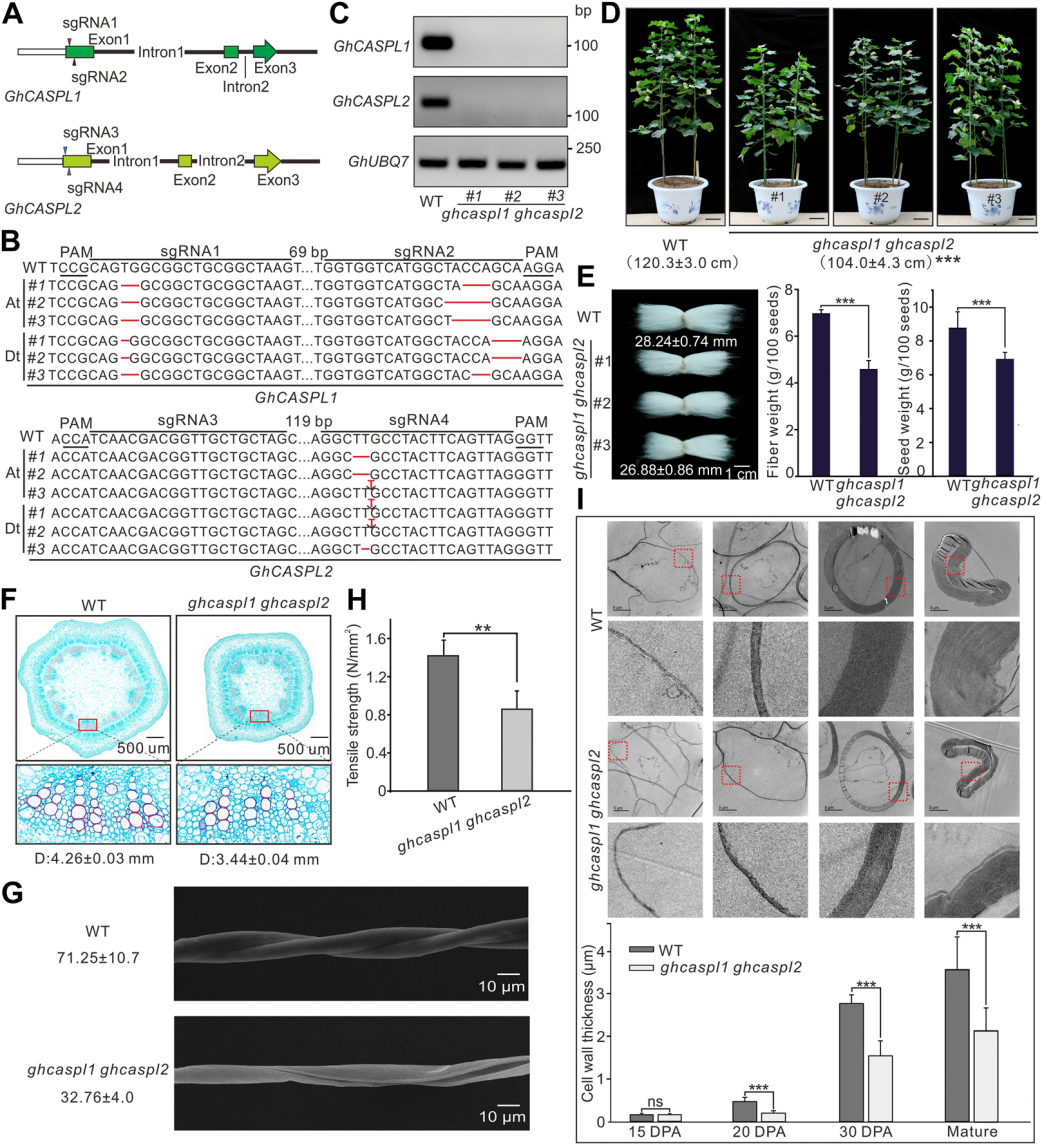
**Genome‐edited** ***ghcaspl1 ghcaspl2*** **knockout mutants show defects in secondary cell wall development in fibers** **(A)** Diagram of the vectors used for knockout of *Gossypium hirsutum CASPARIAN STRIP MEMBRANE DOMAIN‐LIKE1* (*GhCASPL1*) and *GhCASPL2*. The single guide RNAs (sgRNAs) sgRNA1 and sgRNA2 recognized target sites in the At and Dt subgenomes of *GhCASPL1*, while sgRNA3 and sgRNA4 targeted the At and Dt subgenomes of *GhCASPL2*. **(B)** Identification of homozygous knockout mutant lines for *GhCASPL1* and *GhCASPL2*. Each sequence shown in the figure represents the same editing state of the sense and antisense strands in the genome. Three Sanger sequencing reactions were performed on each sample with identical results. The protospacer adjacent motif (PAM) and the sgRNA target sites are indicated by underlining and a horizontal black line, respectively. Black dashed lines with their lengths marked on top of the row indicate that the nucleotide sequences between the two sgRNA sequences were omitted to save space. Nucleotide deletions are represented by red lines, and insertions are shown with red letters. **(C)** Reverse transcription – quantitative polymerase chain reaction analysis of *GhCASPL1* and *GhCASPL2* transcript levels in homozygous knockout mutants. *GhUBQ7* was used as the loading control. **(D)** Representative photographs of the wild type (WT) carrying the empty vector used for transformation and homozygous *ghcaspl1 ghcaspl2* double mutants. Scale bars, 10 cm. **(E)** Analysis of average fiber length (left panel), total fiber weight (middle panel), and seed weight (right panel) in the WT and *ghcaspl1 ghcaspl2* mutant lines. Scale bar, 1 cm. Values are means ± *SD* from three biological replicates. **(F)** Micrographs obtained from transverse sections of stems from 30‐days‐old WT and *ghcaspl1 ghcaspl2* seedlings, stained with safranin O‐Fast Green. The vascular bundles are highlighted and shown in magnified views in the lower panels. Scale bars, 500 μm. **(G)** Analysis of natural twisting in fibers from WT and *ghcaspl1 ghcaspl2* plants, as observed by scanning electron microscopy (SEM). Values (twist numbers/cm) are means ± *SD* obtained from 30 fiber cells. Scale bars, 10 μm. **(H)** Analysis of tensile strength of WT and *ghcaspl1 ghcaspl2* fibers. **(I)** Cell wall thickness in WT and *ghcaspl1 ghcaspl2* fiber cells at different stages of fiber development, as observed by transmission electron microscopy (TEM). Magnified images of the areas highlighted by red squares are shown below each panel. Mean cell wall thickness is reported as means ± *SD* from at least 30 independent cell wall measurements. Days post‐anthesis (DPA) indicates the days after flowering. Scale bars, 5 μm. All significant differences were determined by Student's *t*‐test; **P < 0.01; ***P < 0.001.

To investigate whether GhCASPL1 participates in cellulose biosynthesis, we generated several homozygous mutant lines in cotton by targeting the At and Dt copies of *GhCASPL1* and *GhCASPL2* via CRISPR (clustered regularly interspaced short palindromic repeat)/Cas9 (CRISPR‐associated nuclease 9)‐mediated genome editing (Figure 2A, B). RT‐qPCR analysis showed the absence of transcripts for *GhCASPL1* and *GhCASPL2* in three independent double mutant lines (Figure 2C). Chromatograms from Sanger sequencing confirmed the homozygous state of all double mutant lines (Figure S7). All three homozygous *ghcaspl1 ghcaspl2* mutant lines had a shorter stature than control plants carrying an empty vector (wild type; WT) (104.0 ± 4.3 cm vs. 120.3 ± 3.0 cm, respectively) (Figure 2D). Although all lines successfully completed their vegetative and reproductive cycles (Figure 2D), mature fibers were 5% (Figure 2E, left panel) shorter, with a 35% reduction in weight in the mutants compared to the WT (Figure 2E, middle panel); mutant seeds also weighed 22% less than WT seeds (Figure 2E, right panel).

Analysis of transverse sections of stems from 1‐month‐old cotton seedlings revealed a decrease in the width of vascular bundles in the mutant lines, with a width of 3.44 ± 0.04 mm in the mutants compared to 4.26 ± 0.03 mm in the WT (Figure 2F). We also noticed a 50% decrease in the number of natural twists in mature cotton fibers from the double mutant lines compared to the WT, as observed by scanning electron microscopy (Figure 2G). There was also a 40% decrease in tensile strength in mature mutant fibers compared to the WT (Figure 2H). Transmission electron microscopy (TEM) of ultrathin sections from cotton fiber cells revealed a significant difference in cell wall thickness between WT and *ghcaspl1 ghcaspl2* fibers (Figure 2I, upper panel). While we observed no significant difference in SCW thickness at 15 days‐post‐anthesis (DPA) (corresponding to the SCW transition phase) between the WT and mutants, the SCW of the double mutant lines became significantly thinner when entering the SCW thickening period, from 20–30 DPA until fiber maturity (Figure 2I, lower panel). These results suggest that GhCASPL1 and GhCASPL2 have important effects on cellulose accumulation during SCW formation in cotton fibers.

### The Arabidopsis *caspl1 caspl2* mutant displays cell wall defects in vessel and xylem cells

**Figure 3:**
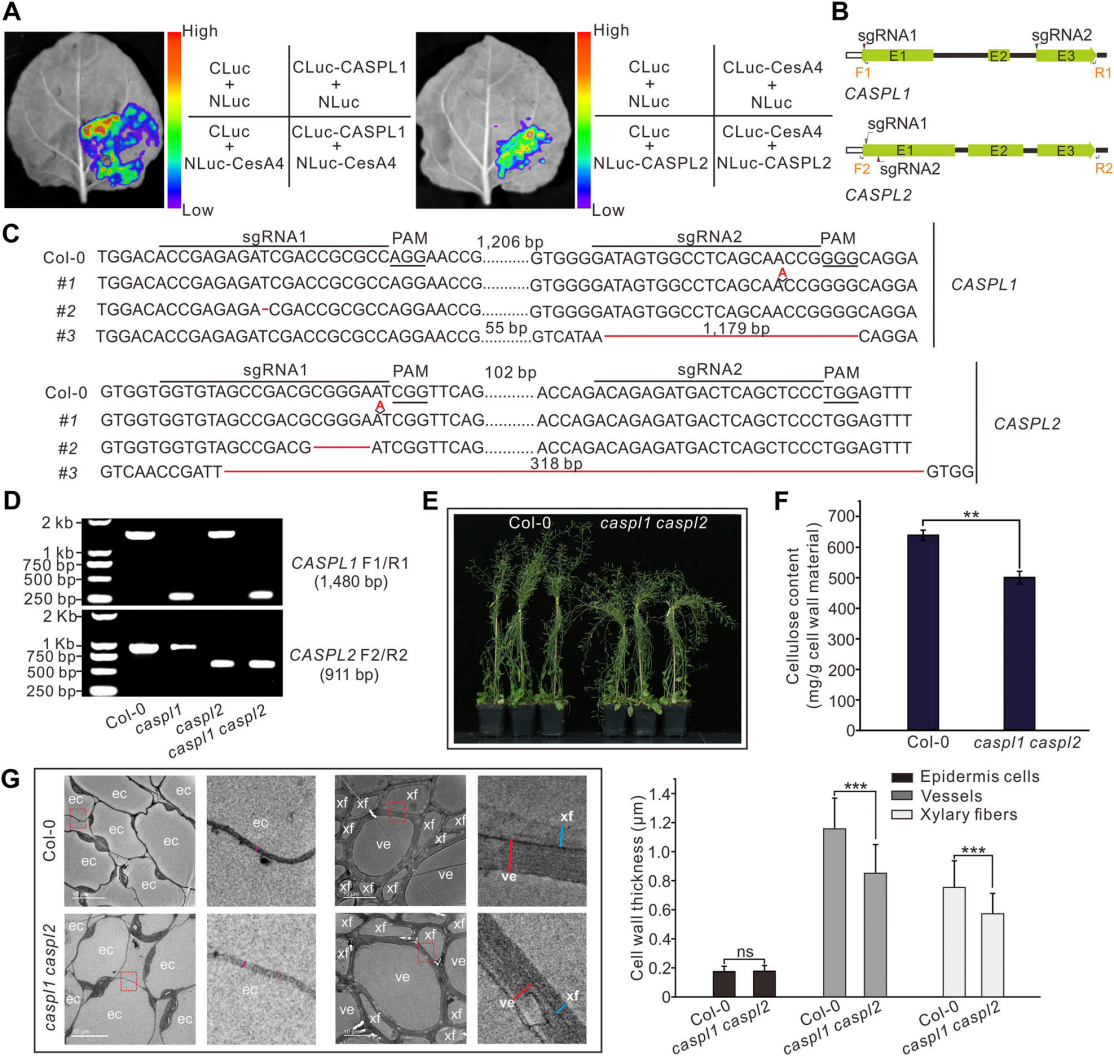
**Arabidopsis** ***caspl1 caspl2*** **double mutants exhibit defects in cell wall thickening in stem vessel and xylem fiber cells** **(A)** Split luciferase assay showing that CASPARIAN STRIP MEMBRANE DOMAIN‐LIKE1 (CASPL1) and CASPL2 interact with Arabidopsis cellulose synthase A4 (CesA4). The constructs *CLuc‐CASPL1*, *NLuc‐CASPL2*, *NLuc*, and *CLuc‐CesA4* were co‐infiltrated into *Nicotiana benthamiana* leaves. Luciferase activity was imaged 48 h later. **(B)** Diagram of the vectors used for genome editing of *CASPL1* and *CASPL2*. E1, E2, and E3 represent the three exons. F1/R1 and F2/R2 indicate the positions of the polymerase chain reaction (PCR) primers. **(C)** Identification of homozygous knockout lines for *CASPL1* and *CASPL2*. The sequences from three independent mutants are shown for each gene. Each sequence shown in the figure represents the same editing state of the sense and antisense strands in the genome. Three Sanger sequencing experiments were performed on each sample with identical results. The protospacer adjacent motif (PAM) region and the single guide RNA (sgRNA) target sites are indicated by underlining and a horizontal black line, respectively. The sequence between the two sgRNAs is not shown. Nucleotide deletions are represented by red lines, and insertions are shown with red letters. **(D)** Identification of knockout mutants by genomic PCR using F1/R1 and F2/R2 primer pairs in wild type (WT: Col‐0), single, and double mutants. The PCR products for *CASPL1* or *CASPL2* are 1,480 or 911 bp in Col‐0 and 301 or 593 bp for *caspl1‐3* or *caspl2‐3*, respectively. The primer positions are shown in (B). **(E)** Representative photographs of 6‐week‐old Arabidopsis WT (Col‐0) and *caspl1 caspl2* plants. **(F)** Cellulose content in the stems of Col‐0 and *caspl1 caspl2* plants. **(G)** Transmission electron micrographs of ultrathin sections from the stems of Col‐0 and *caspl1 caspl2* plants (left panel). ec, epidermal cell; ve, vessels; xf, xylem fiber cells. Magnified images of the areas highlighted with red squares are shown to the right. Mean cell wall thickness is reported on the right, as means ± *SD*. Data represent at least 30 independent cell wall measurements. Significant differences were determined by Student's *t*‐test; **P < 0.01; ***P < 0.001.

Phylogenetic analysis showed that CASPL1 and CASPL2 from Arabidopsis clustered together with GhCASPL1 and GhCASPL2 (Figure S8). Luciferase complementation assays indicated that CASPL1 and CASPL2 interact with CesA4, one of the CSC subunits in Arabidopsis (Figures 3A, S6). We targeted *CASPL1* and *CASPL2* for CRISPR/Cas9‐mediated genome editing (Figure 3B) and obtained homozygous double mutant lines harboring insertions, deletions, or a large deletion in each gene (Figure 3C). We crossed the *caspl1‐3* and *caspl2‐3* single mutants, as they both carried large deletions in their respective genes, to obtain the *caspl1‐3 caspl2‐3* double mutant (*caspl1 caspl2* hereafter) (Figure 3D). The stems of 6‐week‐old double mutant plants appeared to be weaker than those of the Col‐0 control (Figure 3E), which may be attributed to the 22.0% decrease in total cellulose content in mutant stems compared to Col‐0 (Figure 3F). TEM of ultrathin sections of the stem confirmed that the cell walls in both vessels and xylem fibers were significantly thinner in the mutants than in Col‐0, whereas the thickness of epidermal cell walls remained unchanged (Figure 3G). These results indicate that CASPL1 and CASPL2 affect SCW thickening in vessel and xylem fiber cells in Arabidopsis, resembling the activities of GhCASPL1 and GhCASPL2 in cotton.

### Introduction of cotton *GhCASPL1* into Arabidopsis successfully restores the reduced cellulose content of the *caspl1 caspl2* double mutant

**Figure 4:**
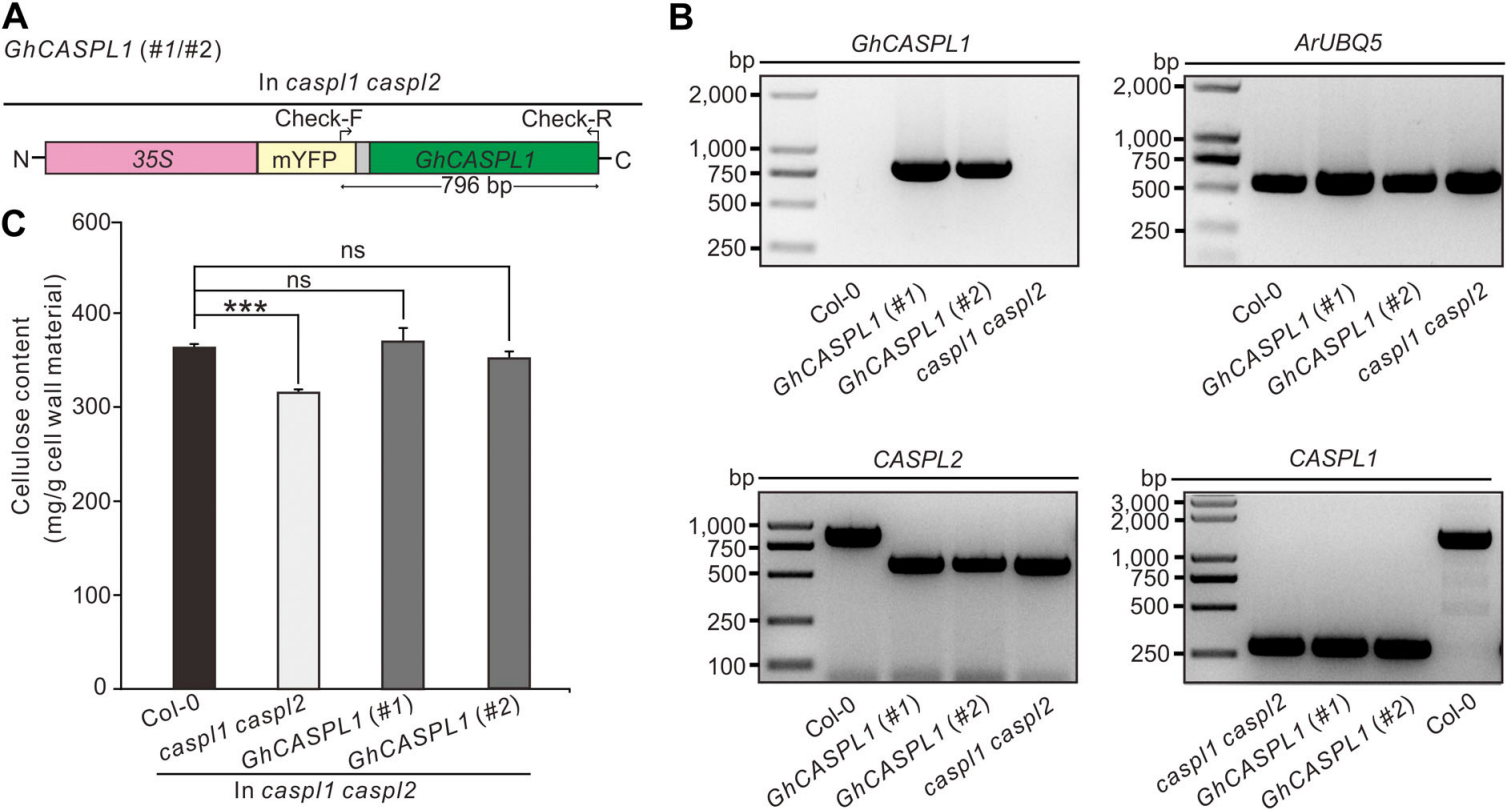
**Introduction of cotton** ***Gossypium hirsutum CASPARIAN STRIP MEMBRANE DOMAIN‐LIKE1 (GhCASPL1*** **) successfully restores the reduced cellulose content of the Arabidopsis** ***caspl1 caspl2*** **double mutant** **(A)** Genomic structure of *GhCASPL1* expressed in the Arabidopsis *caspl1 caspl2* mutant. The promoter is shown in pink, the monomeric yellow fluorescent protein (mYFP) element is shown in yellow, the linker is shown in gray, and the coding sequence of *GhCASPL1* is indicated by a green box. Check‐F and Check‐R primers were employed for the detection of *GhCASPL1*, with arrows and numbers denoting base positions. **(B)** Agarose gel electrophoresis reveals the amplification of an approximately 800 bp fragment using Check‐F and Check‐R primers in GhCASPL1(#1) and GhCASPL1(#2), but not in Col‐0 or *caspl1 caspl2*. Homozygous double knockout lines for *CASPL1* and *CASPL2* were identified, with *ArUBQ5* used as the loading control. **(C)** Cellulose content is restored in the Arabidopsis *caspl1 caspl2* double mutant through heterologous expression of *GhCASPL1*. One‐month‐old Arabidopsis plants grown under normal conditions were used to measure cellulose content. Significant differences were determined by Student's *t*‐test; ***P < 0.001.

To further verify the conservation of CASPL gene function, we created *GhCASPL1*‐expressing plants in the *caspl1 caspl2* mutant background (Figure 4A). We identified the *GhCASPL1* sequence in Arabidopsis plants by RT‐PCR. The target band was detected in plants heterologously expressing *GhCASPL1* but not in WT or *caspl1 caspl2* plants, confirming successful transformation (Figure 4B). We detected a reduction in cellulose contents in *caspl1 caspl2* stems compared to the WT. However, *GhCASPL1*‐expressing plants in the *caspl1 caspl2* background had increased cellulose contents similar to that of the WT (Figure 4C). These findings point to a close relationship between CASPL and cellulose biosynthesis, with GhCASPL1 and its homologous proteins (CASPL1 and CASPL2) in Arabidopsis playing conserved roles in this process.

### CSCs from *ghcaspl1 ghcaspl2* cotton fibers show lower membrane stability and have lower cellulose biosynthesis activity

**Figure 5:**
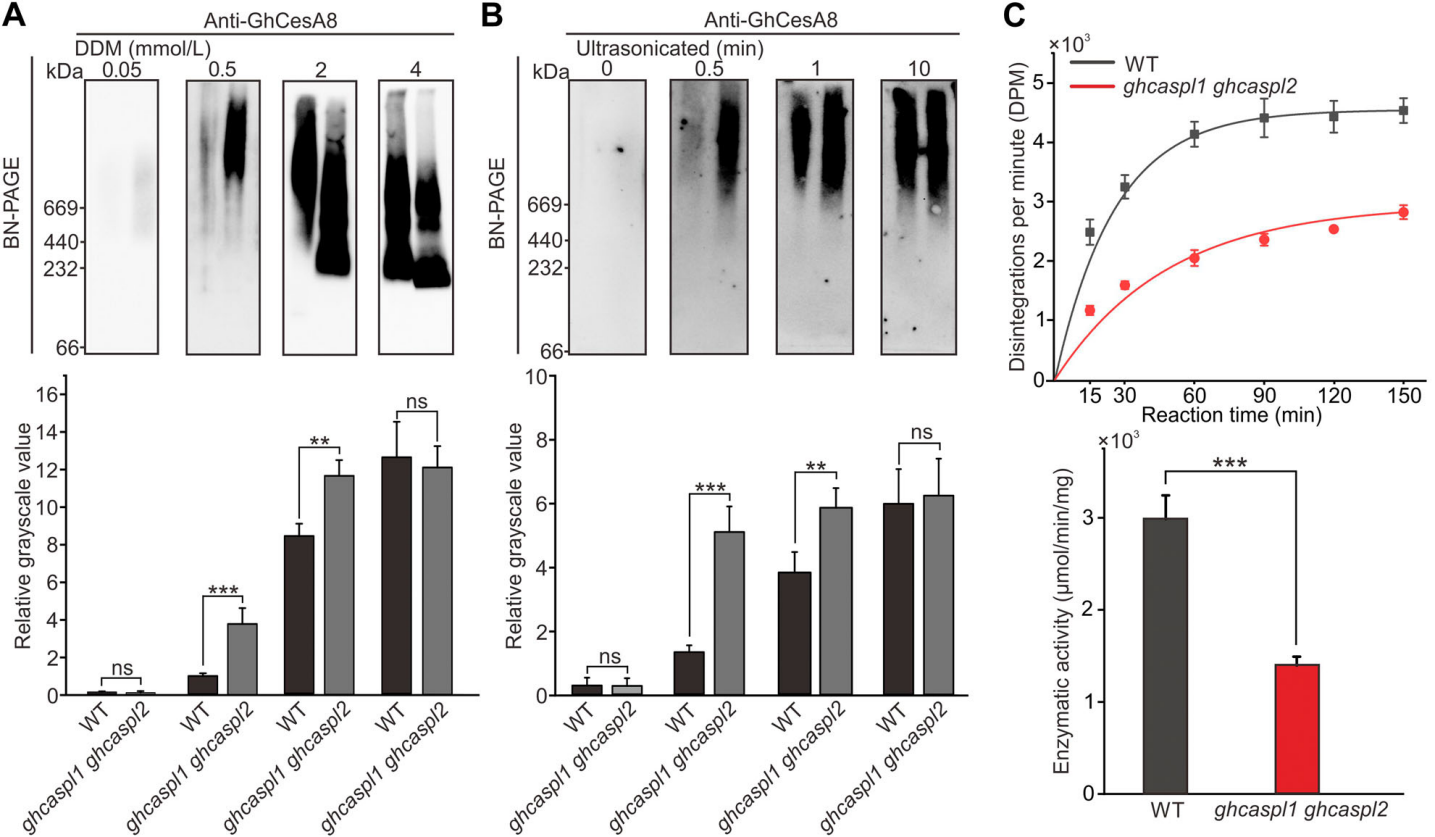
**Compromised cellulose synthase complex (CSC) membrane stability and specific enzyme activity in** ***ghcaspl1 ghcaspl2*** **mutants** **(A)** Analysis of CSC release from membrane fractions of wild‐type (WT) and *ghcaspl1 ghcaspl2* fiber cells treated with different concentrations of n‐dodecyl‐D‐maltoside (DDM). Equal amounts of protein were loaded onto a blue native polyacrylamide gel electrophoresis (BN‐PAGE) gel and subjected to silver staining for quantification. Relative gray scales were measured with ImageJ software. **(B)** Analysis of CSC release from membrane fractions of WT and *ghcaspl1 ghcaspl2* fiber cells after different durations of ultra‐sonication treatment. Equal amounts of protein were loaded onto a BN‐PAGE gel and subjected to silver staining for quantification. **(C)** Cellulose synthase complex enzymatic activity of protein extracts from WT and *ghcaspl1 ghcaspl2* plants. Equal amounts of microsomal fractions prepared from 20 to 25 days‐post‐anthesis cotton fibers were analyzed at optimal pH and temperature. Left, time course of CSC enzymatic reaction using UDP‐[^14^C]‐glucose as a labeled substrate. Right, specific enzyme activity, obtained by calculating the initial slope of the enzymatic reaction from the time course. Values are means ± *SD* from three biological replicates. Significant differences were determined by Student's *t*‐test; **P < 0.01; ***P < 0.001.

The integrity of the CSC at the membrane is crucial for SCW formation in cotton fibers (Wen et al., 2022). To assess the stability of the CSC at the membrane, we prepared microsomal fractions from 20 to 25 DPA WT and *ghcaspl1 ghcaspl2* fibers and incubated them with different concentrations of the detergent n‐dodecyl β‐d‐maltoside (DDM) to release membrane‐bound proteins (Figure S9, left panel). We then separated the solubilized proteins by blue native polyacrylamide gel electrophoresis (BN‐PAGE), transferred them to a membrane, and probed them by immunoblotting with anti‐GhCesA8 antibodies (Wen et al., 2022). Using a DDM concentration of 0.05 mmol/L, no CesA8 was detectable in either WT or mutant samples, indicating that the CSC stayed attached to the membrane at this low detergent concentration (Figure 5A). When we increased the DDM concentration to 0.5 mmol/L, much more GhCesA8 became released from microsomes prepared from *ghcaspl1 ghcaspl2* fibers, with some GhCesA8 also released from WT samples (Figure 5A, upper panel). As the DDM concentration increased to 2 or 4 mmol/L, even more GhCesA8 was released from microsomes isolated from WT and *ghcaspl1 ghcaspl2* fibers, with WT samples reaching the same GhCesA8 signal strength as that of the double mutant when treated with 4 mmol/L DDM. The relative grayscale (hybridization signal) ratio between mutant and WT samples was 3.90, 1.38, and 0.96 at DDM concentrations of 0.5, 2, and 4 mmol/L, respectively (Figure 5A, lower panel), suggesting that CSCs from mutant fiber cell membranes were more unstable and more easily washed away by the detergent than those from the WT.

To further examine CSC stability, we subjected these microsomal fractions to ultrasonication treatment (Figure S9, right panel). Upon 0.5 min of sonication, much more GhCseA8 was released from microsomal samples of the *ghcaspl1 ghcaspl2* mutant than the WT (Figure 5B, upper panel). With a longer duration of ultrasonication treatment, the amount of GhCesA8 released from *ghcaspl1 ghcaspl2* microsomes remained high, whereas that released from WT microsomes progressively rose with longer treatment, reaching levels similar to that of *ghcaspl1 ghcaspl2* after 10 min (Figure 5B, upper panel). The relative grayscale ratio between mutant and WT samples was 3.87, 1.53, and 1.04 in samples sonicated for 0.5, 1, and 10 min, respectively (Figure 5B, lower panel). These results indicate that the stability of the CSC on the fiber membrane is reduced in the *ghcaspl1 ghcaspl2* double mutant compared to the WT.

In a CSC enzymatic activity assay, microsomal fractions prepared from the WT accumulated more cellulose product in a shorter time than those prepared from an equal amount of *ghcaspl1 ghcaspl2* microsomal fractions (Figure 5C, upper panel). The specific CSC enzymatic activity of the mutant, as determined by calculating the initial velocity of the enzymatic reaction, was approximately half that of the WT (Figure 5C, lower panel). Thus, the lower CSC membrane stability observed in *ghcaspl1 ghcaspl2* affects its enzymatic activity, resulting in compromised SCW biosynthesis in fibers.

### GhCASPL1 binds to phosphatidic acid to maintain the anchoring of the CSC to the PM

**Figure 6:**
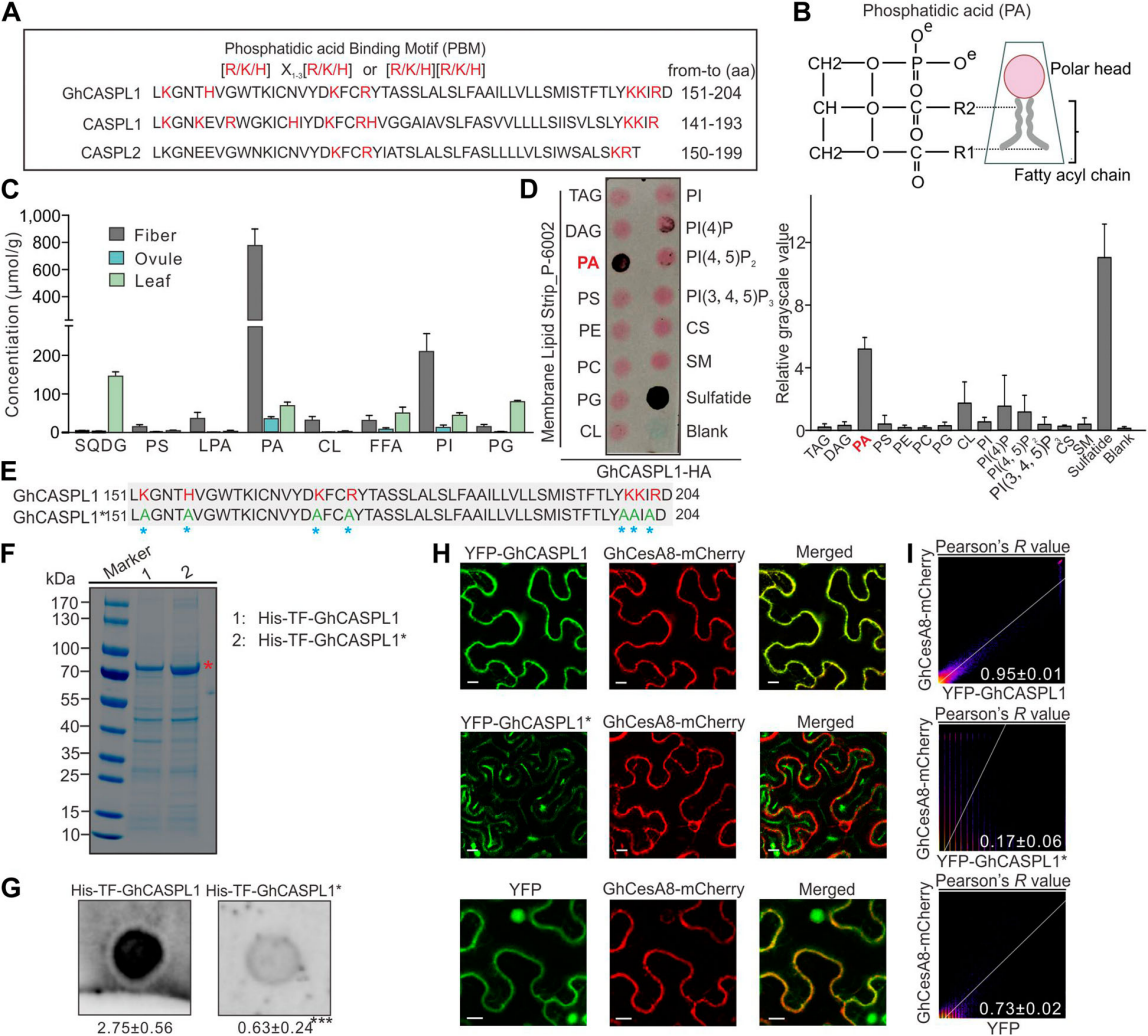
***Gossypium hirsutum*** **CASPARIAN STRIP MEMBRANE DOMAIN‐LIKE1 (GhCASPL1) contributes to secondary cell wall formation in cotton fibers by binding to phosphatidic acid (PA) and anchoring the CSC to the plasma membrane** **(A)** Amino acid sequence alignment of GhCASPL1, CASPL1, and CASPL2 showing the classic PA‐binding motif. Positively charged amino acids are shown in red. **(B)** Diagram showing the structure of PA. The red head is the polar head; the gray lines represent the fatty‐acid chain. **(C)** Analysis of phospholipid contents during secondary wall development in fibers, ovules, and mature leaf tissues. CL, cardiolipin; FFA, free fatty acids; LPA, lyso‐PA; PA, phosphatidic acid; PG, phosphatidylglycerol; PI, phosphatidylinositol; PS, phosphatidylserine; SQDG, sulfoquinovosyl diglyceride. **(D)** Membrane lipid overlay assay using GhCASPL1‐HA (hemagglutinin) protein extracts (left), and quantification of binding (right) based on grayscale values, as measured using ImageJ software. Values are means ± *SD* from three biological replicates. PI (4)P, phosphatidylcholine (4) phosphate; PI (4,5)P_2_, phosphatidylinositol (4) bisphosphate; PI(3,4,5)P_3_, phosphatidylinositol (3,4,5) trisphosphate; CS, cholesterol; SM, sphingomyelin; sulfatide, 3‐sulfogalactosylceramide. **(E)** Multiple sequence alignment of the PA‐binding motif of GhCASPL1 following mutation, resulting in the creation of a new variant named GhCASPL1*. The mutated amino acid, indicated by a blue asterisk, was consistently changed to alanine (A). **(F)** Coomassie blue staining of purified His‐TF‐GhCASPL1 and His‐TF‐GhCASPL1* recombinant proteins (~75 kDa), with asterisks marking the location of the target protein. **(G)** *In vitro* membrane lipid overlay assay of His‐TF‐GhCASPL1 and His‐TF‐GhCASPL1* with PA. Quantitative analysis of grayscale values in the dot blots was performed. Values represent the mean of three independent measurements, with error bars representing *SD*. Significant differences were determined by Student's *t*‐test; ***P < 0.001. **(H)** The yellow fluorescent protein (YFP)‐GhCASPL1 fusion protein co‐localizes with GhCesA8‐mCherry in the plasma membrane, while the YFP‐GhCASPL1* fusion protein with the mutated PA‐binding motif does not co‐localize with GhCesA8‐mCherry. YFP and GhCesA8‐mCherry were used as the negative controls. Scale bars, 10 μm. **(I)** Analysis of Pearson's *r* values to assess the colocalization of the fusion proteins in H. A strong correlation was observed between YFP‐GhCASPL1 and GhCesA8‐mCherry (*r* = 0.95 ± 0.01), whereas YFP‐GhCASPL1* and GhCesA8‐mCherry showed a notably weaker correlation (*r* = 0.17 ± 0.06). The negative control of YFP and GhCesA8‐mCherry showed a weaker correlation (*r* = 0.73 ± 0.02).

We identified putative PA‐binding motifs in GhCASPL1 and its homologs in Arabidopsis (Figure 6A). PA is composed of a polar head with two negative charges and two fatty acyl tails (Figure 6B), which may interact with membrane‐anchored proteins to tether them to the membrane and promote protein complex formation or stability (Liu et al., 2013; Yao and Xue, 2018). We therefore reasoned that the PA content of fiber cells might be different from that of ovule and leaf tissues, which might help explain the lower CSC stability observed in the mutant. To investigate this issue, we performed phospholipomics analysis of cotton fibers and ovules harvested during the SCW thickening stage and from leaf tissue. We measured the eight types of phospholipids analyzed in these tissues and determined that PA, which accumulated to high levels in fiber cells, was much less abundant in samples prepared from developing leaves or ovules (Figure 6C). The contents of sulfoquinovosyl diglyceride (SQDG) and phosphatidylglycerol (PG), which are present in photosynthetic membranes, were much higher in leaves than in fibers or ovule tissue (Figure 6C).

In a lipid dot‐blot assay, among all lipids present on the membrane, only PA and sulfatide showed strong binding to recombinant GhCASPL1, with phosphatidylinositol 4‐phosphate (PI(4)P), phosphatidylinositol 4,5‐bisphosphate (PI(4,5)P), and cardiolipin (CL) showing modest binding activities (Figure 6D). Sulfatide was discovered in human brains and is not found in plants (Kushi et al., 1996; Blomqvist et al., 2021). Subsequently, we expressed and purified GhCASPL1 and GhCASPL1* with a mutation in the PA‐binding motif *in vitro* (Figure 6E). GhCASPL1 is a four‐spanning membrane protein known for its extremely low solubility. To improve its solubility, we modified GhCASPL1 by adding chaperone TF and His tags to the N‐terminus, resulting in the fusion protein His‐TF‐GhCASPL1/GhCASPL1*, with an approximate molecular weight of 75 kDa. The recombinant proteins His‐TF‐GhCASPL1 and His‐TF‐GhCASPL1* were successfully induced, confirmed with immunoblot (Figure S10A, B), and obtained following large‐scale expression, affinity purification, and gel filtration chromatography (Figure 6F). In an *in vitro* membrane lipid overlay assay, His‐TF‐GhCASPL1 exhibited high‐affinity binding to PA (grayscale values 2.7 ± 0.56), whereas His‐TF‐GhCASPL1* with a mutation in the PA‐binding motif showed negligible binding to PA (grayscale values 0.63 ± 0.24) (Figure 6G).

Confocal microscopy revealed the colocalization of YFP‐GhCASPL1 and GhCesA8‐mCherry in the PM. The mutation of the PA‐binding site in GhCASPL1 resulted in the loss of its colocalization with GhCesA8, and it failed to localize to the PM (Figure 6H). A strong correlation of *r* = 0.95 ± 0.01 was observed between YFP‐GhCASPL1 and GhCesA8‐mCherry, whereas YFP‐GhCASPL1* and GhCesA8‐mCherry exhibited a significantly weaker correlation of *r* = 0.17 ± 0.06 (Figure 6I, upper and middle panel). By contrast, the negative control, YFP and GhCesA8‐mCherry, showed a weaker correlation of *r* = 0.73 ± 0.02 (Figure 6I, lower panel). These results suggest that the binding of GhCASPL1 to PA modulates the stability of the CSC on the PM.

**Figure 7:**
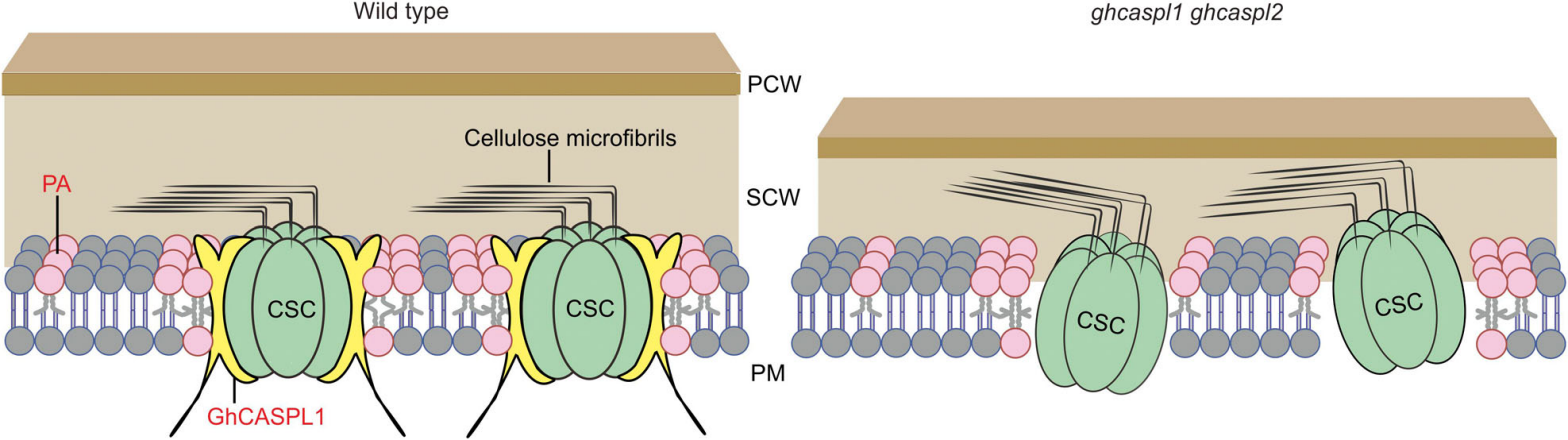
**A proposed model showing how** ***Gossypium hirsutum*** **CASPARIAN STRIP MEMBRANE DOMAIN‐LIKE1 (GhCASPL1) may help maintain cellulose synthase complex (CSC) stability on the plasma membrane** In the wild type, GhCASPL1 interacts with phosphatidic acid (PA) and the CSC, which helps stabilize the CSC to ensure efficient biosynthesis of cellulose microfibrils in the secondary cell wall (SCW). In *ghcaspl1 ghcaspl2*, the CSC is loosely associated with the cell membrane, resulting in decreased specific enzymatic activity and diminished SCW biosynthesis.

We propose a model to explain how GhCASPL1 and PA jointly help stabilize the CSC structure to ensure efficient biosynthesis of cellulose microfibrils in the SCW (Figure 7, left panel). In the absence of GhCASPL1 and GhCASPL2, the CSC becomes more loosely associated with the cell membrane, resulting in lower specific enzymatic activity and compromised SCW biosynthesis (Figure 7, right panel).

## DISCUSSION

Cotton fiber cells accumulate large amounts of cellulose, rather than lignin or hemicellulose, in a short period of time during SCW formation (Huang et al., 2021; Wen et al., 2023). This accumulation depends on the enzymatic activity of the CSC located on the PM (Scavuzzo‐Duggan et al., 2018; Wen et al., 2022). Thus, maintaining the stability of the CSC on the PM is essential for rapid cellulose biosynthesis. CASPLs are membrane‐spanning proteins, but little is known about their functions (Roppolo et al., 2014). Here, we demonstrated that the expression patterns of *GhCASPL1* and *GhCesA8* are similar during SCW formation (Figure 1B, D, E) and that their encoded proteins interact, suggesting that GhCASPL1 may contribute to the deposition of cell wall materials. MARVEL domain‐containing proteins may be involved in protein dimerization (Yaffe et al., 2012), promote membrane localization, or form microdomains (Magal et al., 2009). Here, we demonstrated that GhCASPL1 interacts with CSC subunits (Figure 1J–L) and that the loss of GhCASPL1 and GhCASPL2 function was accompanied by significantly reduced CSC membrane stability and a concomitant decrease in cellulose biosynthesis activity (Figure 5). With four transmembrane helices (Figure 1F, G), the relatively small protein GhCASPL1 may be highly hydrophobic and perform membrane‐anchoring functions. GhCASPL1 localizes to the PM (Figure 1H, I). These findings support the idea that CASPL proteins act as membrane scaffolds involved in cell wall modification (Roppolo et al., 2011; Roppolo et al., 2014).

Mutating the Arabidopsis homologs *CASPL1* and *CASPL2* resulted in cell wall defects in vessels and xylem fiber cells (Figure 3G), similar to those observed in fibers of the *ghcaspl1 ghcaspl2* mutant. However, in contrast to the *ghcaspl1 ghcaspl2* double mutant, the height of Arabidopsis *caspl1 caspl2* plants was not significantly different from the WT. This difference may be attributed to the inherent differences in stem composition between woody and herbaceous plants. Changes in xylem and fiber cells may have more pronounced effects on the growth and development of woody plants, whereas their influence on herbaceous stems would be relatively minimal (Tong et al., 2021).

The CSC is responsible for SCW biosynthesis and is composed of three CesAs (Desprez et al., 2007; Wen et al., 2022), with each CesA possessing seven transmembrane domains embedded in the lipid membrane (Zhang et al., 2021). The CSC is located in lateral patches in the PM that are resistant to detergents and exhibit similar properties to lipid rafts in animals (Bessueille et al., 2009). To date, only sterol‐ and GPI‐anchoring proteins, such as SETHs (Lalanne et al., 2004), COBRA (Roudier et al., 2005), and SALT OVERLY SENSITIVE5 (SOS5) (Harpaz‐Saad et al., 2011), were shown to interact with different CSC subunits during PCW formation to modulate their activity. Here, quantitative lipidomics analysis showed that fiber cells harvested during the SCW thickening period contained higher amounts of PA than other known phospholipids (Figure 6C). In addition, the PA content was much higher in cotton fiber cells than in mature leaves and ovules (Figure 6C). PA is characterized by a small polar head with two thick acyl tails composed of conical anionic lipids to prevent tight packing with the head groups of neighboring phospholipids, thus contributing to membrane rearrangement by generating negative membrane curvature (Zhukovsky et al., 2019; Wang et al., 2024). We discovered that GhCASPL1 strongly binds to PA but not to other lipids (Figure 6D, G), pointing to a potential interaction between these two partners in tethering the CSC onto the PM for efficient cellulose biosynthesis and SCW thickening. Disruption of the PA‐binding site in GhCASPL1 resulted in its loss of colocalization with GhCesA8, and the mutant GhCASPL1 protein could not localize to the PM (Figure 6H, I). Nevertheless, mutation of *ghcaspl1 ghcaspl2* does not affect the localization of GhCesA8 on the PM (Figure S11). Thus, we present a working model describing the joint roles of GhCASPL1 and PA in maintaining a stable CSC structure in the PM to ensure the efficient biosynthesis of cellulose microfibrils in the SCW (Figure 7).

In summary, we discovered a new CSC interactor, GhCASPL1, and characterized its contribution to SCW formation via its interactions with the CSC and the membrane lipid PA. These findings enhance our understanding of the mechanisms controlling SCW formation in cotton fibers, which has implications for breeding cotton varieties with high‐quality fiber in the future. Further research is required to identify additional plasma membrane‐anchoring proteins associated with the CSC to explore the role of the membrane lipid environment in regulating cellulose microfibril biosynthesis in the SCW by the CSC.

## MATERIALS AND METHODS

### Plant materials and growth conditions

Wild‐type and mutant cotton (*G. hirsutum*) plants were grown in fully automated walk‐in growth chambers (29 ± 2°C; 14‐h‐light/10‐h‐dark photoperiod). Cotton fibers were collected at different stages of boll development relative to DPA. Fibers at the indicated DPA were isolated by scratching the ovule quickly with tweezers for RT‐qPCR, microsomal fractionation, immunoblotting analysis, and TEM of ultrathin sections. Samples from 1‐month‐old seedlings were collected for paraffin sectioning and microscopy. To measure mature cotton fiber length, the fibers (approximately 80 g for each genotype) were tested at the Center of Cotton Fiber Quality Inspection and Testing, Chinese Ministry of Agriculture (Anyang, Henan Province, China). *Nicotiana benthamiana* and *Arabidopsis thaliana* (Col‐0 accession) plants were grown at 22 ± 2°C under a 16‐h‐light/8‐h‐dark photoperiod. Six‐week‐old Arabidopsis plants were used for PCR, cellulose content analysis, paraffin sectioning, ultrathin sectioning, and microscopy.

### Co‐immunoprecipitation and MS

For IP–MS, more than 100 g (fresh weight) of fiber cells (at 20 DPA) were harvested and ground to a fine powder in liquid nitrogen for microsomal extraction. All steps after harvesting were performed on ice. The fiber powder was resuspended in cold MOPS buffer (100 mmol/L MOPS, pH 7.0, 2 mmol/L ethylenediaminetetraacetic acid, 2 mmol/L ethyleneglycoltetraacetic acid, and Protease Inhibitor Cocktail Tablets (Roche)) and mixed as gently as possible to form a rough pulp. The mixture was filtered through three layers of Miracloth (Millipore, USA), and the supernatant was collected into a 50‐mL conical centrifuge tube to remove cell debris by centrifugation at 10,000 × *g* for 15 min at 4°C. The resulting supernatant was gently transferred to a fast ultracentrifugation tube and centrifuged at 100,000 × *g* for 2 h at 4°C using an SW28 rotor. After centrifugation, the centrifuge tube was carefully placed on ice, the supernatant was discarded, and the pellet was gently resuspended in an equal volume of dilution buffer (10 mmol/L phosphate‐buffered saline (PBS), pH 7.4 with 1× Complete Protease Inhibitor Cocktail (Roche)). Microsomal membranes (500 μL at 10 μg/μL) were incubated for 30 min at 4°C in the presence of 2 mmol/L DDM with continuous stirring. Membrane debris was removed by centrifugation at 100,000 × *g* for 30 min at 4°C, and the supernatant containing DDM‐solubilized membrane proteins was used for immunoprecipitation.

Magnetic Dynabeads (30 μL, Thermo Scientific, USA) were washed three times with 1 mL PBS (pH 7.5) for 10 min each time. After adding specific antibodies against GhCesA8, GhCesA4, or GhCesA7 (~10 μg each), the beads were incubated on a rotator at room temperature for 1 h. The DDM‐solubilized membrane proteins were added to the beads with the corresponding antibodies, and the mixture was incubated at 4°C overnight with gentle rotation. The beads were captured on a magnetic stand and washed three times with washing buffer (50 mmol/L Tris‐HCl, 150 mmol/L NaCl, and 0.5% (v/v) Tween‐20, pH 7.4). Specific proteins bound to the beads were eluted by adding 40 μL protein lysate buffer (50 mmol/L Tris‐HCl pH 6.8, 2% (w/v) sodium dodecyl sulfate (SDS), 10% (v/v) glycerol, and 1% (w/v) β‐mercaptoethanol). The samples were incubated at 95°C for 5 min and separated by SDS‐PAGE (polyacrylamide gel electrophoresis) for MS. Rabbit immunoglobulin G (IgG) was used as a negative control. MS was performed as previously described (Wen et al., 2022).

### RNA extraction and gene expression analysis

Total RNA was extracted from cotton samples using an RNAprep pure Plant Plus Kit (Tian‐gen Biotech, Beijing, China). Total RNA (1 μg) was used as a template for first‐strand complementary DNA (cDNA) synthesis with Oligo dT Primers and EasyScript One‐Step genomic DNA (gDNA) Removal and cDNA Synthesis SuperMix (TransGen Biotech). The resulting cDNA was diluted three times in sterile water and used as a template for qPCR with PerfectStart Green qPCR SuperMix (TransGen Biotech, Beijing, China). *GhUBQ7* was used as the internal control.

### Sequence analysis

The *GhCASPL1* and *GhCASPL2* genomic sequences and GhCASPL1 and GhCASPL2 protein sequences were downloaded from the cotton genome database (http://www.cottongen.org) with the updated cotton genome sequence (Huang et al., 2020). Transmembrane domains in GhCASPL1 were predicted using the T‐Coffee package (https://tcoffee.crg.eu/apps/tcoffee/all.html) (Chang et al., 2012). *CASPL* gene sequences from Arabidopsis were downloaded from The Arabidopsis Information Resource using GhCASPL1 as a query (TAIR, https://www.arabidopsis.org). The sequences of GhCASPL1 and related proteins were aligned using Clustal Omega with default parameters. A neighbor‐joining phylogenetic tree of GhCASPL1 and homologs in *G. hirsutum* and Arabidopsis was constructed using MEGAX software.

### Immunoblotting

Denatured proteins were loaded onto 4% (w/v) to 20% (w/v) SDS‐PAGE gradient gels (ACE Biotechnology, Cat. No. ET15420Gel) and separated at 120 V for 2 h. A wet transfer was performed at 100 V for 2 h in ice‐cold transfer buffer (20 mmol/L Tris‐HCl, 40 mmol/L glycine, and 20% (v/v) methanol). The membranes were blocked in 5% (w/v) nonfat dry milk with Tween 20 and Tris‐buffered saline (TTBS) buffer at room temperature for 3 h on a shaking table (100 rpm). The blocked membranes were incubated in milk with TTBS buffer with the corresponding primary antibody at 4°C overnight with gentle rotation. The following antibodies were used: anti‐Actin (Abbkine, Wuhan, China, Cat. No. ABL1050, 1:10,000 dilution), anti‐HA (Mabnus, Wuhan, China, Cat. No. GS20004, 1:5,000 dilution), anti‐histone H3 (Affinity Biosciences, America, Cat. No. AF0863, 1:2,000 dilution), anti‐UGPase (YNK BIOTECH, Suzhou, China, Cat. No. YKZP902, 1:10,000 dilution), anti‐H^+^‐ATPase (Agrisera, Sweden, Cat. No. AS07260, 1:3,000 dilution), and anti‐GhCesA8 (our laboratory, 1:10,000 dilution). Peroxidase‐conjugated goat anti‐rabbit (ZB‐2301, ZSGB‐BIO) or goat anti‐mouse antibodies (ZB‐2305, ZSGB‐BIO) were used as secondary antibodies.

### Subcellular localization

The full‐length coding sequences of *GhCesA8*, *GhCASPL1*, GhCASPL1*, and *PIP2A* were amplified from *G. hirsutum* or Arabidopsis cDNA and cloned into the vector, respectively, as before. The primers are listed in Table S3. All vectors were transformed into *N. benthamiana* leaves via *Agrobacterium tumefaciens* strain GV3101‐mediated transformation. The fluorescent signal was recorded on the third day after infiltration under a Leica stereo microscope (TCS, SP8).

### Protein–protein interaction assays

For the Co‐IP assay, DDM‐solubilized membrane proteins were isolated from cotton fibers at 20 DPA as described above. The protein extracts were then incubated overnight at 4°C with gentle rotation with anti‐HA magnetic beads (Mabnus, Cat. No. M7004). Immune complexes were collected by centrifugation and washed six times in wash buffer (50 mmol/L Tris‐HCl, 150 mmol/L NaCl, and 0.5% (v/v) Tween‐20, pH 7.4). Co‐IP proteins that bound to the beads were eluted with 30 μL protein lysate buffer, incubated at 95°C for 5 min, and separated by SDS‐PAGE for immunoblotting with anti‐HA and anti‐GhCesA8 antibodies.

For the luciferase (LUC) assays, the full‐length coding sequences of *GhCesA8*, *GhCASPL1*, *CesA4*, *CASPL1*, and *CASPL2* were individually amplified from *G. hirsutum* or Arabidopsis cDNA and cloned into the NLuc‐X or Clux‐X vector. The primers used for cloning are listed in Table S3. The other details were as previously described (Gao et al., 2022).

A DUAL‐membrane yeast two‐hybrid (MY2H) assay was conducted following the manufacturer's instructions (Clontech, America). Briefly, the full‐length coding sequences of *GhCesA4*, *GhCesA7*, *GhCesA8*, and *GhCASPL1* were individually cloned into the ppR3‐N and pBT3‐SUC vectors and transformed in the appropriate pairs into yeast (*Saccharomyces cerevisiae*) competent cells (strain NYM51). Positive transformants were selected based on growth on synthetic defined medium lacking Trp, and the presence of plasmids was confirmed by PCR. Independent positive yeast colonies were diluted in 600 μL distilled water and serially diluted in 10‐fold increments before spotting 10 μL of each dilution onto synthetic defined/−Ade/−His/−Trp/−Leu solid medium to test for interactions.

### CRISPR/Cas9‐mediated genome editing

CRISPR‐Cas9‐mediated editing of *GhCASPL1* and *GhCASPL2* was conducted by Towin Biotechnology (Wuhan, China) as previously reported (Ran et al., 2013; Wang et al., 2018; Zhang et al., 2023). Methods for the identification of transgenic lines were previously described (Wen et al., 2022). A transgenic cotton line (*proGhCASPL1*:*GhCASPL1‐HA*) harboring a transgene consisting of the endogenous *GhCASPL1* promoter driving the expression of *GhCASPL1* encoding the HA tag was also generated by Towin Biotechnology (Wuhan, China).

### Cell wall analysis

Dried fibers were coated with gold using an ion sputtering coating instrument (Pengde Technology, China) and observed under a scanning electron microscope (Hitachi, Tokyo, Japan) operated at 20 kV. An electronic universal testing machine is used to test various mechanical properties such as metal or non‐metallic materials in tension, compression, bending, and shearing. The Shiyanjia Lab platform in conjunction with the electronic universal testing machine (CMT6103/ZWICK) was utilized to conduct mechanics performance testing on both WT and mutant mature cotton fibers. The procedure involved selecting materials of equal length and quality for testing; at least 12 bundles of cotton fibers of equal quality per sample were used for testing. Initially, a suitable clamp for cotton fibers was chosen for use with a 1KN sensor. The upper and lower clamps were then employed to secure the test sample, with an inlet force of 0.01 N and a tensile rate of 10 mm/min. The test was initiated by clicking to start the process, and the changes in force with displacement during the tensile test were subsequently recorded. To analyze fiber thickness, ultrathin sections of the cell walls of cotton fibers were generated and observed TEM as previously described (Wen et al., 2022).

To measure cellulose content, a 50‐mm piece from the primary inflorescence stem of an Arabidopsis plant was harvested from 5 mm above the rosette level and stored at −80°C prior to analysis. The material was thawed and ground to a powder in liquid nitrogen. Cellulose content was measured using a Cellulose (CLL) Content Assay Kit (Beijing Boxbio Science & Technology, Cat. No. AKSU007M). In detail, cell wall materials were extracted and dried to a constant weight to calculate the cellulose content. During this process, cellulose is broken down into β‐D‐glucose under acidic conditions and dehydrated under strong acidic conditions to produce β‐furfural compounds. These compounds are then dehydrated and condensed with anthrone to create blue‐green furfural derivatives with a distinct absorption peak at 620 nm. By tracking the changes in absorbance values, the cellulose content could be precisely measured.

### Blue native PAGE and cellulose synthase activity assay

Blue native PAGE and cellulose synthase activity assays were performed as described (Wen et al., 2022) with a few modifications. The protein concentration of the microsomal membrane suspension was first determined using a Pierce BCA Protein Assay Kit (Thermo). For ultrasonication, membrane microsome fractions with equal amounts of protein were placed in a Covaris M220 Focused Ultrasonicator controlled with SonoLab7.2 software for the appropriate time (0.5, 1 and 10 min) (Li et al., 2023). Microsomal membrane extracts containing equal amounts of protein were also incubated for 30 min at 4°C in the presence of the appropriate concentration (0.05, 0.5, 2, and 4 mmol/L) of DDM with continuous stirring. All membrane fragments were removed by centrifugation at 100,000 × *g* for 30 min at 4°C, and the supernatant containing solubilized membrane proteins was used for BN‐PAGE experiments.

*In vitro* cellulose biosynthesis was performed with microsomal membrane proteins extracted from WT and *ghcaspl1 ghcaspl2* plants as previously described using UDP‐[^14^C]‐glucose as a substrate (Brown et al., 2012; Wen et al., 2022).

### Lipidomic analysis

Freshly collected cotton fibers and ovules (24 DPA) and leaf tissues were inactivated with hot isopropanol as previously described (Liu et al., 2020; Miao et al., 2023). Extraction solvent (chloroform: methanol: 300 mmol/L ammonium acetate in water [30:41.5:3.5, v/v/v]) was added to the samples, followed by incubation at 4°C for 30 min at 1500 rpm. The supernatant was transferred to a fresh tube. The inactivation (72°C) and extraction steps were repeated once. The lipid extracts from both rounds of extraction were pooled and dried in a SpeedVac (Genevac, UK). Total protein content in the dried pellet was measured using a Pierce BCA Protein Assay Kit (Thermo). Lipid extracts were stored at −80°C until liquid chromatography (LC)–MS analysis.

All lipidomic analyses were conducted at LipidALL Technologies on a Shimadzu Nexera 20AD‐HPLC instrument coupled with Sciex QTRAP 6500 PLUS as previously reported (Liu et al., 2020). For normal‐phase analysis of polar lipids, individual species were separated using a TUP‐HB silica column (i.d. 150 × 2.1 mm, 3 µm) under the following conditions: mobile phase A (chloroform: methanol: ammonium hydroxide, 89.5: 10: 0.5) and mobile phase B (chloroform: methanol: ammonium hydroxide: water, 55: 39: 0.5: 5.5). Individual lipid species were quantified in reference to spiked internal standards, including d31‐PS(d31‐16:0/18:1), d7‐PA33:1(15:0/18:1), d7‐PG33:1(15:0/18:1), d5‐CL72:8(18:2)4, DMPS, DMPA, DMPG, from Avanti Polar Lipids (Alabaster, AL) and LIPID MAPS. Dioctanoyl phosphatidylinositol (PI) (16:0‐PI) was purchased from Echelon Biosciences, Inc. (Salt Lake City, UT, USA) and used together with d7‐PI33:1(15:0/18:1) (Avanti Polar Lipids) for PI quantitation. Free fatty acids were quantitated using d31‐16:0 (Sigma‐Aldrich, Germany).

### Protein–lipid overlay assay

Membrane lipid strips (P‐6002) containing 15 purified membrane lipids were purchased from Echelon Biosciences. The lipid overlay assay was performed as previously described with some modifications (Yang et al., 2022). First, the strips were incubated with 10 mL 3% (w/v) fatty‐acid free ovalbumin (A5253; Sigma) in PBS‐T blocking buffer (PBS and 0.1% (v/v) Tween 20; pH 7.4) for 3 h at room temperature. The strips were then incubated with a final concentration of 1 μg/mL DDM‐solubilized membrane proteins from HA lines in 3 mL of PBS‐T with 3% (w/v) fatty‐acid free bovine serum albumin (BSA) at room temperature for 3 h. The strips were washed six times for 10 min each in PBS‐T and incubated with anti‐HA primary antibody (diluted 1:4000) in 3 mL of 3% (w/v) fatty‐acid free BSA in PBS‐T at 4°C overnight. Subsequently, the strips were incubated with secondary anti‐mouse antibodies (ZB‐2305, ZSGB‐BIO, diluted 1:10,000) in blocking buffer for 2 h at room temperature, followed by three 10‐min washes with PBS‐T. Finally, the membranes were processed for chemiluminescence detection.

### Recombinant protein expression and purification

*GhCASPL1* and GhCASPL1* (with a mutated PA‐binding site) were cloned and assembled into pCold‐TF, which includes a 50 kDa trigger factor chaperone and a 6 × His tag. All constructs were expressed in *Escherichia coli* strain BL21 (DE3).

To purify soluble proteins, whole cell lysates (50 mmol/L Tris‐HCl, 300 mmol/L NaCl, 20 mmol/L imidazole, pH 7.5) were centrifuged at 13,000 × *g*. The suspension was collected and purified on a HisTrap Fast Flow Column (GE). Target recombinant proteins were eluted with His‐elution buffer (50 mmol/L Tris‐HCl, 300 mmol/L NaCl, 300 mmol/L imidazole, pH 8.0). Subsequently, the proteins underwent further purification by size exclusion using a Superdex 200 column. The fractions were collected, concentrated on a 10 kDa molecular weight cutoff Centricon Centrifugal Filter Unit (Millipore), dialyzed, and identified by SDS‐PAGE.

Sequence information about *GhCASPL*s, *GhCesA*s, *CASPL*s, and *CesA*s mentioned in this paper can be found in the cotton database (http://202.114.67.150/genomebrowser/index.html) and TAIR (https://www.arabidopsis.org/index.jsp), respectively. The accession numbers are as follows: *GhCASPL1* (Ghi_A02G01601, Ghi_D02G02196), *GhCASPL2* (Ghi_A09G14651, Ghi_D09G11011), *GhCesA8* (Ghi_A10G02401, Ghi_D10G01681), *GhCesA4* (Ghi_A08G02596, Ghi_D08G02486), *GhCesA7* (Ghi_A05G02931, Ghi_D05G00431), *CASPL1* (At4g15610), *CASPL2* (At3g06390), and *CesA4* (At5g44030).

## CONFLICTS OF INTEREST

The authors declare no conflict of interest.

## AUTHOR CONTRIBUTIONS

L.Z. and Y.X.Z. designed the experiments; L.Z., X.C., and X.P.W. performed the experiments; L.Z., K.W., and Y.F.Z. analyzed the data; L.Z. wrote the manuscript; Y.X.Z. revised the manuscript. All authors read and approved the manuscript.

## Supporting information

Additional Supporting Information may be found online in the supporting information tab for this article: http://onlinelibrary.wiley.com/doi/10.1111/jipb.13777/suppinfo

**Figure S1.** Multiple sequence alignment of *Gossypium hirsutum* CASPARIAN STRIP MEMBRANE DOMAIN‐LIKE1 (GhCASPL1) homologous proteins
**Figure S2.** Sequence identities and expression levels in different cotton tissues for all potential *Gossypium hirsutum CASPARIAN STRIP MEMBRANE DOMAIN‐LIKE1* (*GhCASPL*) genes
**Figure S3.** *Gossypium hirsutum* CASPARIAN STRIP MEMBRANE DOMAIN‐LIKE2 (GhCASPL2) is localized in the plasma membrane
**Figure S4.** Identification of *proGhCASPL1*:*GhCASPL1*‐*HA* transgenic strain in cotton
**Figure S5.** Protein fractions from membrane microsomes with the anti‐hemagglutinin (anti‐HA) antibody were used to perform immunoprecipitation – mass spectrometry (IP‐MS) assay
**Figure S6.** Split luciferase assay showing that *Gossypium hirsutum* CASPARIAN STRIP MEMBRANE DOMAIN‐LIKE1 (GhCASPL1) interacts with *Gossypium hirsutum* cellulose synthase A4 (GhCesA4) (A) and GhCesA7 (B) respectively
**Figure S7.** Sanger sequencing data to confirm the homozygous situation of the double mutant cotton lines
**Figure S8.** Phylogenetic analysis of *Gossypium hirsutum* CASPARIAN STRIP MEMBRANE DOMAIN‐LIKE1 (GhCASPL1) homologous protein from cotton and *Arabidopsis thaliana*
**Figure S9.** Protein silver staining was performed on wild‐type (WT) and mutant microsomes after solubilization in n‐dodecyl β‐d‐maltoside (DDM) (A) and ultrasonic treatment (B)
**Figure S10.** Verification of His‐TF‐GhCASPL1/His‐TF‐GhCASPL1* recombinant protein expression level
**Figure S11.** Mutation of *ghcaspl1 ghcaspl2* does not affect the localization of *Gossypium hirsutum* cellulose synthase A8 (GhCesA8) on the plasma membrane
**Figure S12.** The mutant plants did not show altered sensitivity to the cellulose synthesis inhibitor isoxaben since it produced similar effect on either wild‐type (WT) or *caspl1 caspl2* mutant plants
**Table S1.** Immunoprecipitation – mass spectrometry (IP–MS) information of 91 shared IP proteins against *Gossypium hirsutum* cellulose synthase GhCesA4, GhCesA7, and GhCesA8 antibodies
**Table S2.** The expression patterns of 91 shared *Gossypium hirsutum cellulose synthases* – immunoprecipitation (*GhCesAs*‐IP) proteins across different tissues of cotton
**Table S3.** Primers used in this study
**Table S4.** Differentially expressed genes of wild‐type and g*hcaspl1 ghcaspl2* mutants in the cotton fiber during secondary cell wall (SCW) development
