# Supplementary material for: GhCASPL1 regulates secondary cell wall thickening in cotton fibers by stabilizing the cellulose synthase complex on the plasma membrane
Source: J Integr Plant Biol. 2024 Sep 24;66(12):2632–47. doi: 10.1111/jipb.13777 (PMC11622535; doi:10.1111/jipb.13777)
Supplement: Supplementary file 1 — Figure S1. Multiple sequence alignment of Gossypium hirsutum CASPARIAN STRIP MEMBRANE DOMAIN‐LIKE1 (GhCASPL1) homologous proteins Figure S2. Sequence identities and expression levels in different cotton tissues for all potential Gossypium hirsutum CASPARIAN STRIP MEMBRANE DOMAIN‐LIKE1 (GhCASPL) genes Figure S3. Gossypium hirsutum CASPARIAN STRIP MEMBRANE DOMAIN‐LIKE2 (GhCASPL2) is localized in the plasma membrane Figure S4. Identification of proGhCASPL1:GhCASPL1‐HA transgenic strain in cotton Figure S5. Protein fractions from membrane microsomes with the anti‐hemagglutinin (anti‐HA) antibody were used to perform immunoprecipitation – mass spectrometry (IP‐MS) assay Figure S6. Split luciferase assay showing that Gossypium hirsutum CASPARIAN STRIP MEMBRANE DOMAIN‐LIKE1 (GhCASPL1) interacts with Gossypium hirsutum cellulose synthase A4 (GhCesA4) (A) and GhCesA7 (B) respectively Figure S7. Sanger sequencing data to confirm the homozygous situation of the double mutant cotton lines Figure S8. Phylogenetic analysis of Gossypium hirsutum CASPARIAN STRIP MEMBRANE DOMAIN‐LIKE1 (GhCASPL1) homologous protein from cotton and Arabidopsis thaliana Figure S9. Protein silver staining was performed on wild‐type (WT) and mutant microsomes after solubilization in n‐dodecyl β‐d‐maltoside (DDM) (A) and ultrasonic treatment (B) Figure S10. Verification of His‐TF‐GhCASPL1/His‐TF‐GhCASPL1* recombinant protein expression level Figure S11. Mutation of ghcaspl1 ghcaspl2 does not affect the localization of Gossypium hirsutum cellulose synthase A8 (GhCesA8) on the plasma membrane Figure S12. The mutant plants did not show altered sensitivity to the cellulose synthesis inhibitor isoxaben since it produced similar effect on either wild‐type (WT) or caspl1 caspl2 mutant plants Table S1. Immunoprecipitation – mass spectrometry (IP–MS) information of 91 shared IP proteins against Gossypium hirsutum cellulose synthase GhCesA4, GhCesA7, and GhCesA8 antibodies Table S2. The expression patterns of 91 sh [file JIPB-66-2632-s001.docx]

**Supplementary Figure 1**


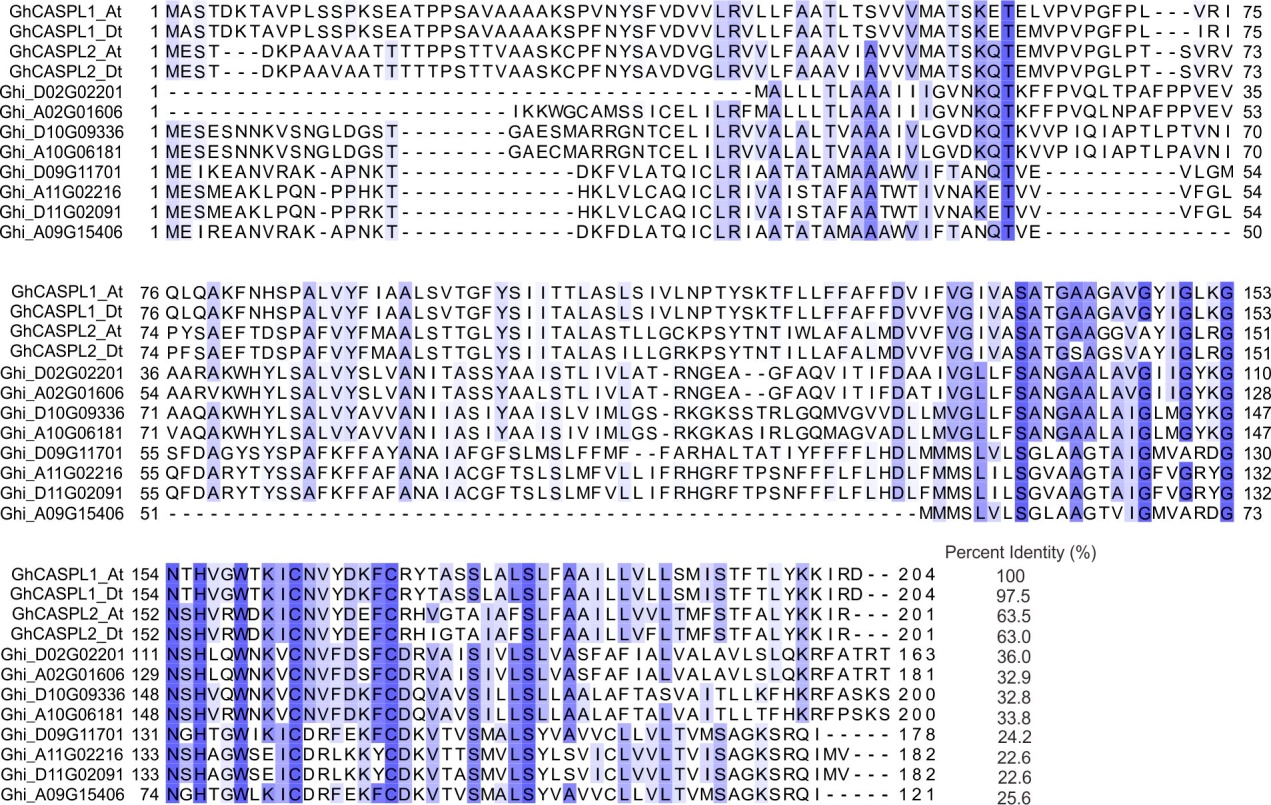


**Supplementary Figure 1 Multiple sequence alignment of GhCASPL1 homologous proteins.**

The alignment was produced with Clustal Omega using default parameters.

**Supplementary Figure 2**


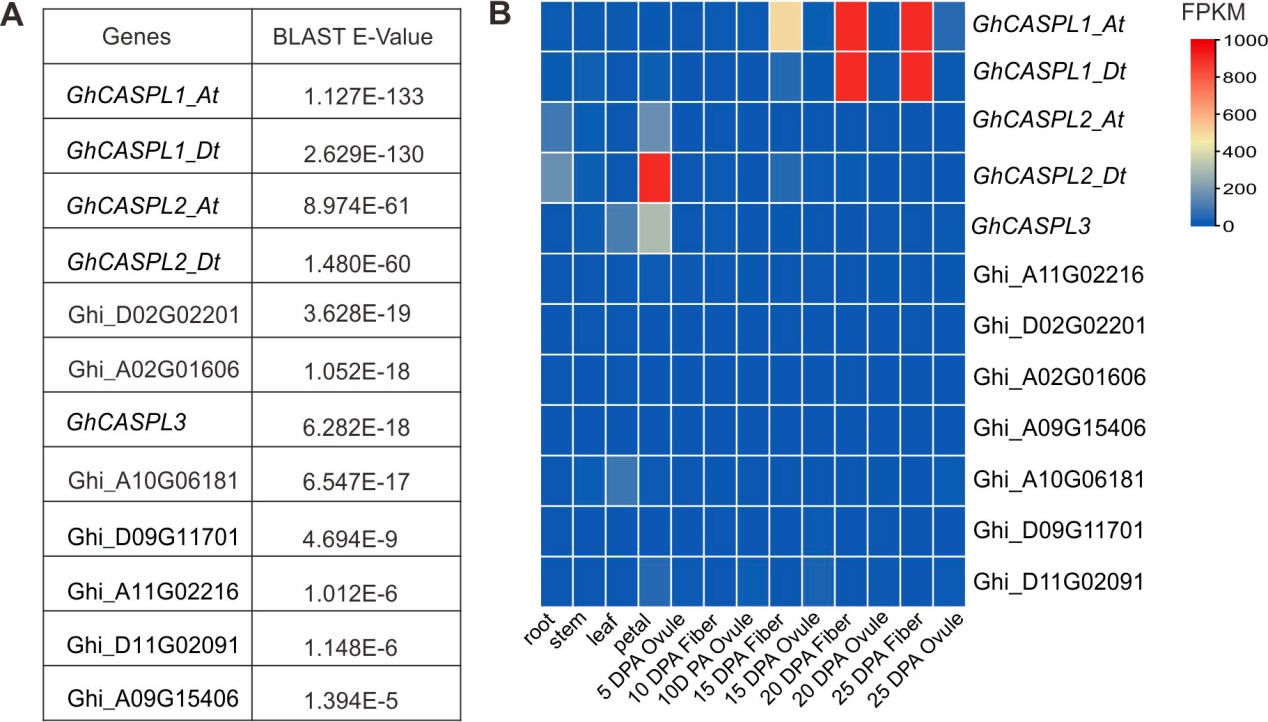


**Supplementary Figure 2 Sequence identities and expression levels in different cotton tissues for all potential *GhCASPL* genes.**

A, The GhCASPL1 pro tein sequence was used to blast in the Cottongene database with default parameters, a total of 12 homologous genes were found. B, The expression levels of homologous genes in different tissues of cotton, the original data comes from the CottonMD database.

**Supplementary Figure 3**


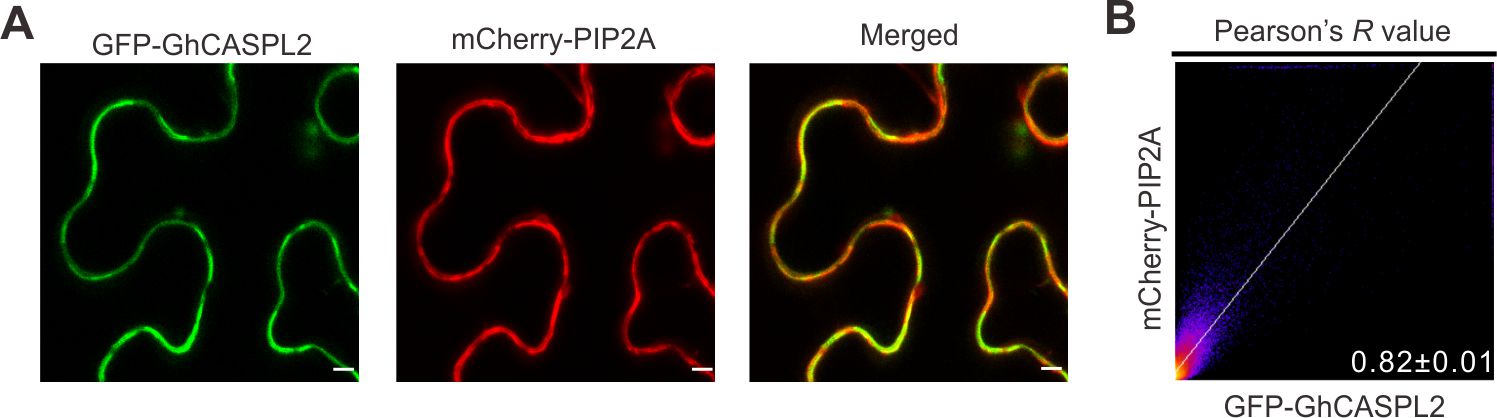


**Supplementary Figure 3 GhCASPL2** **is localized in the plasma membrane.**

A, Confocal microscopy indicating that the YFP-GhCASPL2 fusion protein co-localized with mCherry-PIP2A, a plasma membrane marker, implying that YFP-GhCASPL2 is indeed located in the plasma membrane.

B, Analysis of Pearson’s *r* values to assess colocalization of the fusion proteins in A. A strong correlation was observed between YFP-GhCASPL2 and mCherry-PIP2A (*r* = 0.82 ± 0.01).

**Supplementary Figure 4**


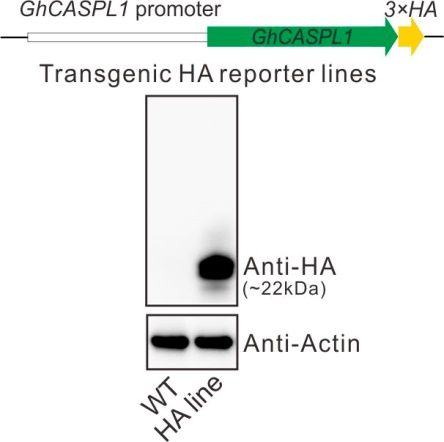


**Supplementary Figure 4 Identification of *proGhCASPL1*:*GhCASPL1*-*HA* transgenic strain in cotton.**

Upper panel, Schematic of HA labeled transgenic lines. Lower panel, Immunoblot analysis of anti-HA antibody in total cotton fiber proteins.

**Supplementary Figure 5**


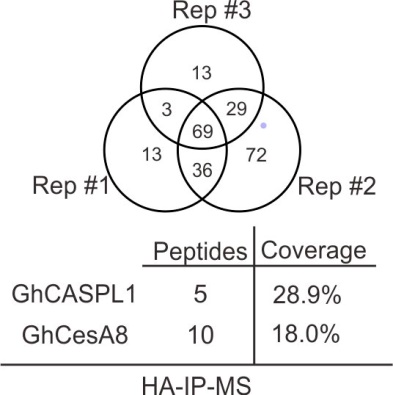


**Supplementary Figure 5 Protein fractions from membrane microsomes with the anti-HA antibody were used to perform IP-MS assay.**

The peptides and coverage information of GhCASPL1 and GhCesA8 were averaged from three independent experiments.

**Supplementary Figure 6**

**
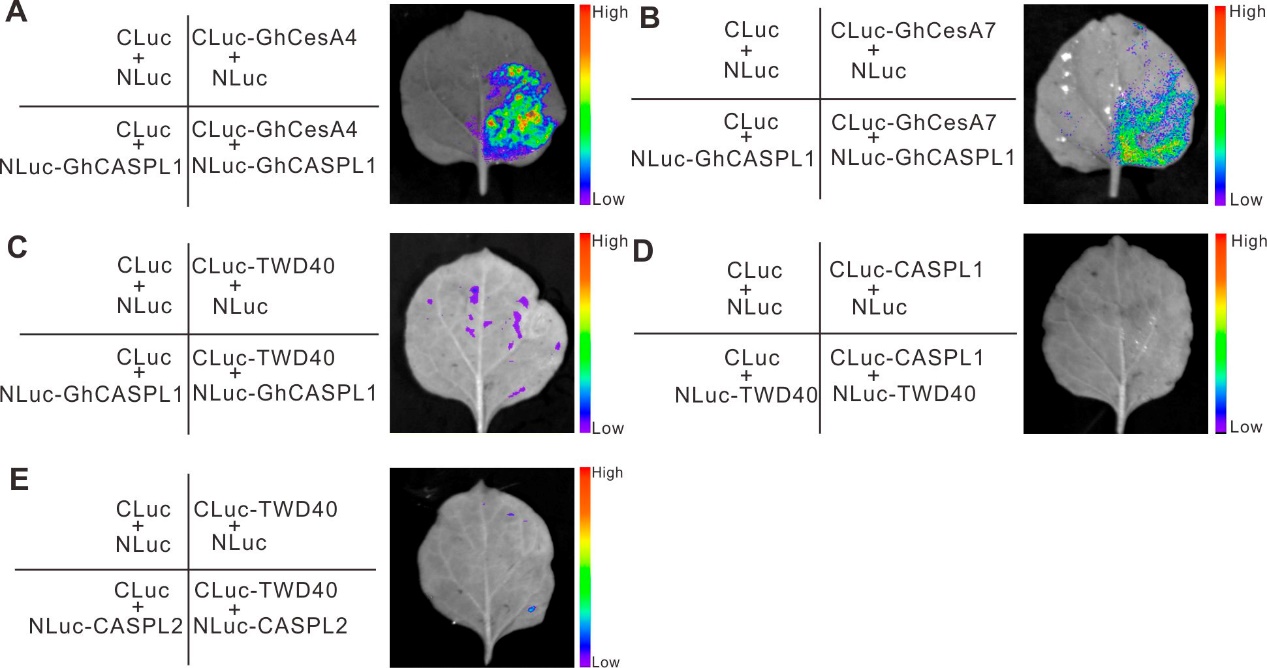
**

**Supplementary Figure 6 Split luciferase assay showing that GhCASPL1 interacts with GhCesA4 (A) and GhCesA7 (B) respectively.**

C, D and E indicate that GhCASPL1, CASPL1 and CASPL2 do not exhibit interactions with TWD40 in plants as negative control, respectively. The constructs CLuc-GhCesA4, CLuc-GhCesA7, NLuc-GhCASPL1, CLuc-CASPL1, NLuc-CASPL2 and CLuc/NLuc-TWD40 were co-expressed in *N. benthamiana* leaves, which were imaged for luciferase activity 48 h later.

**Supplementary Figure 7**

**
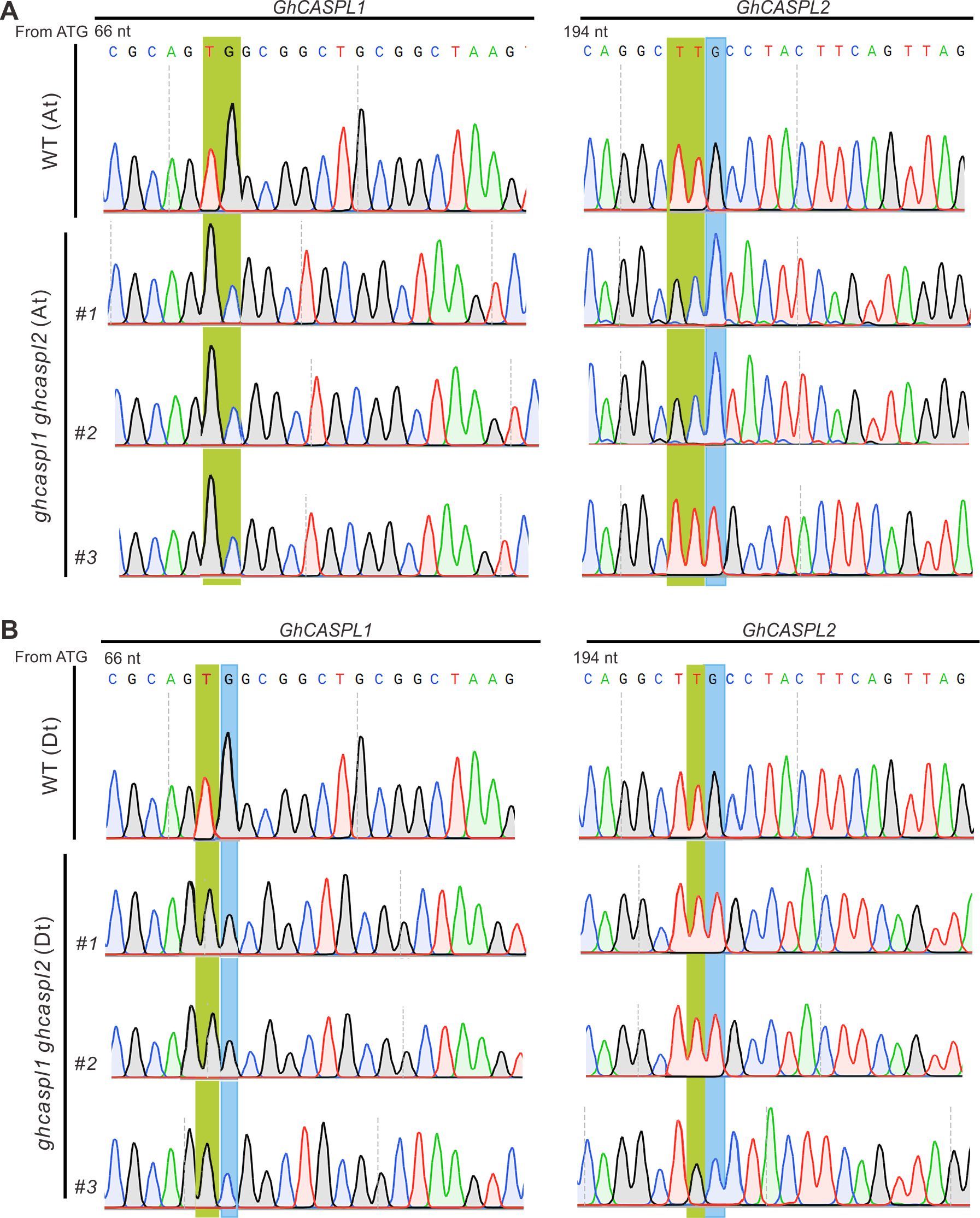
**

**Supplementary Figure 7 Sanger sequencing data to confirm the homozygous situation of the double mutant cotton lines.**

A, represents the situation on the At subgenome; B is what happens on the Dt subgenome. The light green and blue boxes indicate the site where the editing occurred.

**Supplementary Figure 8**


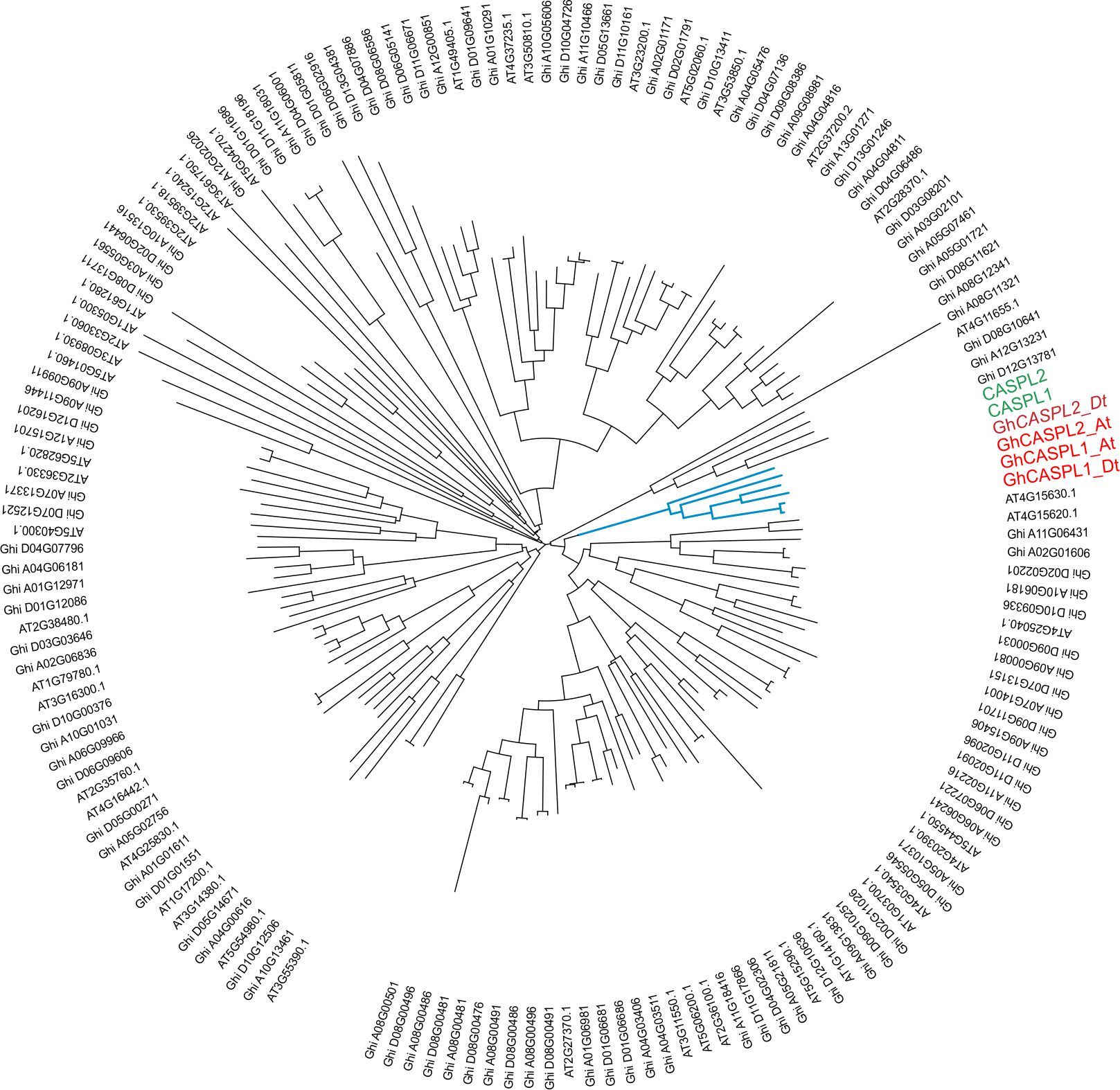


**Supplementary Figure 8 Phylogenetic analysis of GhCASPL1 homologous protein from cotton and *Arabidopsis thaliana*.**

The phylogenetic tree was constructed with the neighbor-joining (NJ) method by MEGAX.

**Supplementary Figure 9**


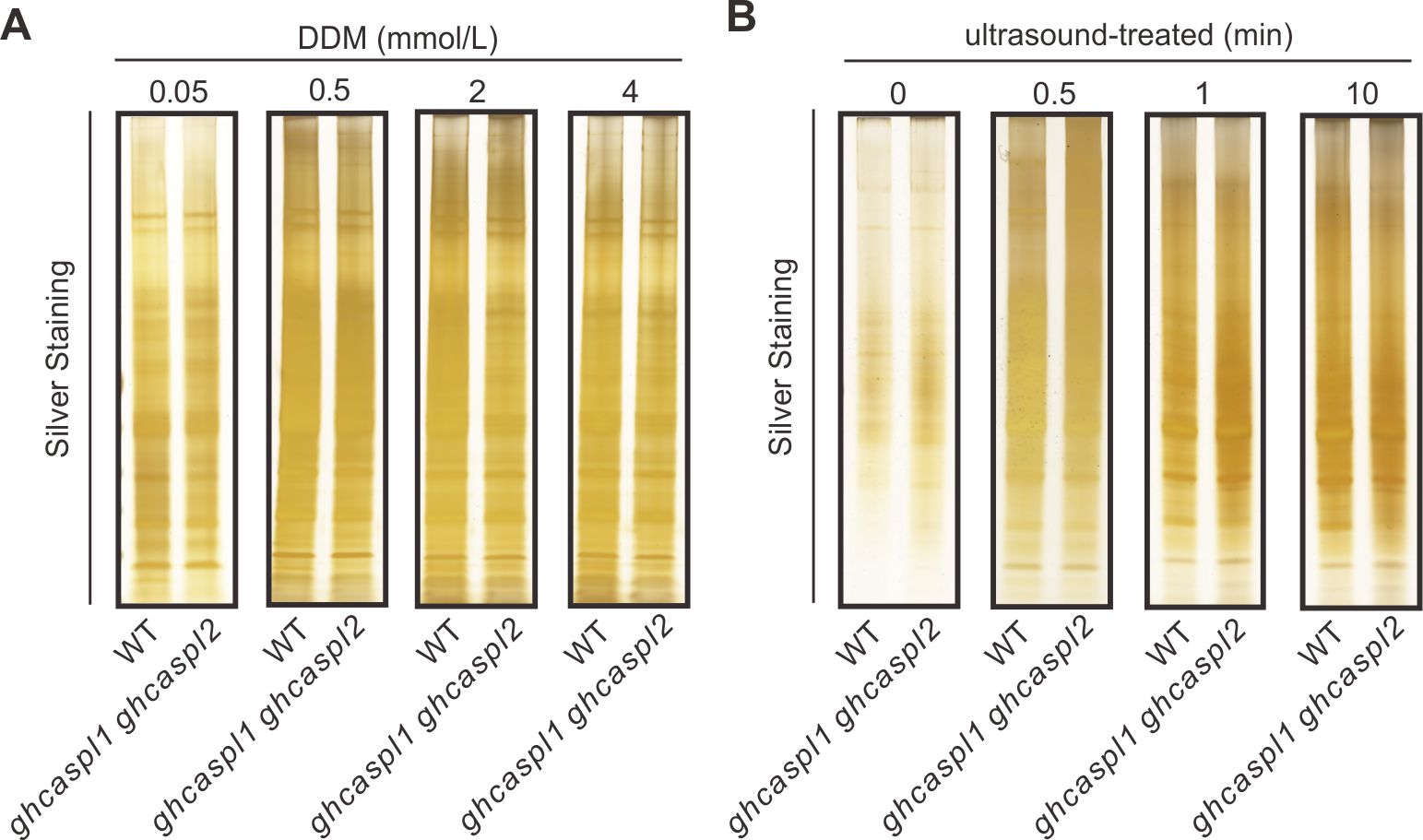


**Supplementary Figure 9 Protein silver staining was performed on WT and mutant microsomes** **after solubilization in DDM (A) and ultrasonic treatment (B).**

Equal amounts of total protein were sequentially eluted from cotton fiber microsomal extracts.

**Supplementary Figure 10**


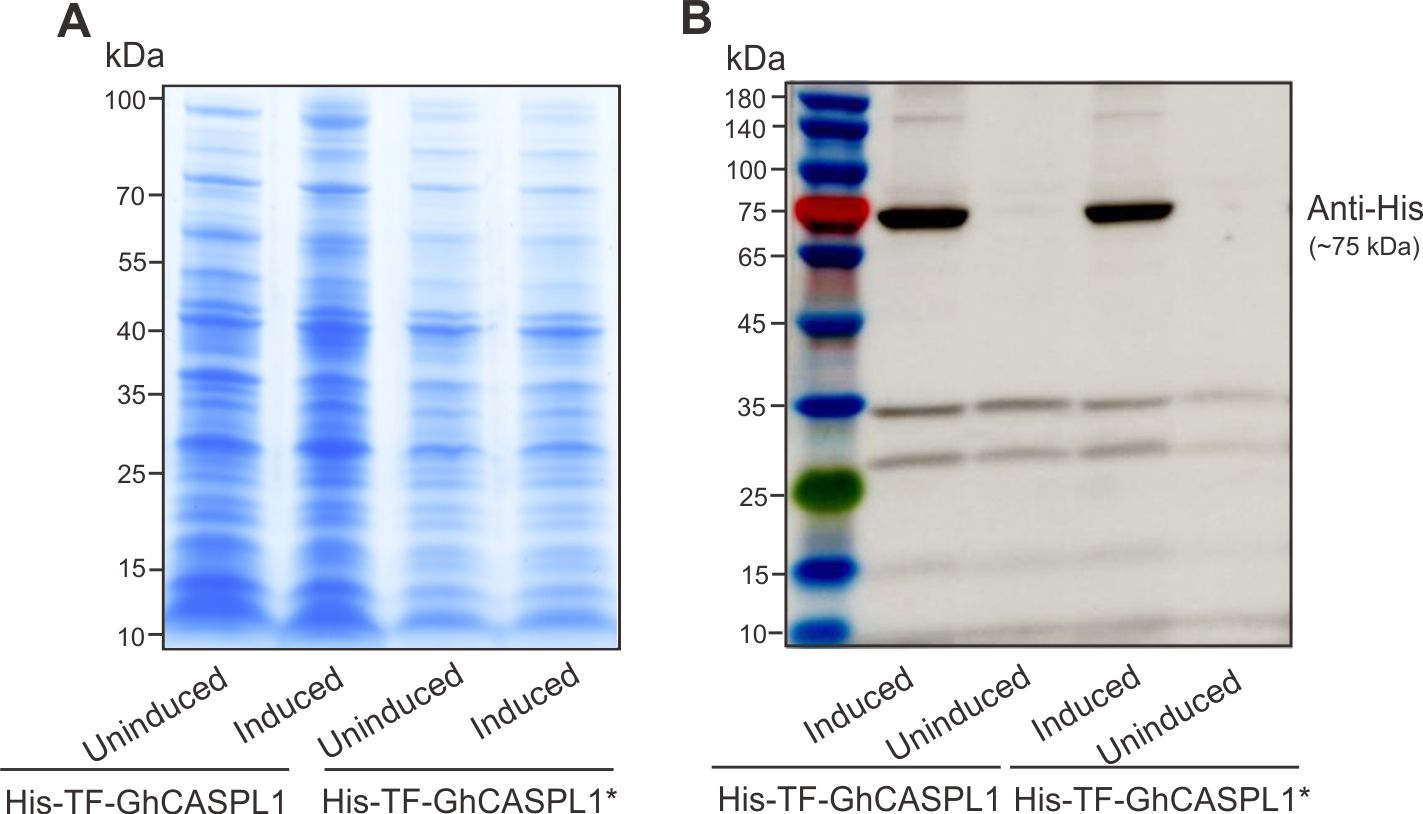


**Supplementary Figure 10 Verification of His-TF-GhCASPL1/** **His-TF-GhCASPL1* recombinant protein expression level.**

A, Recombinant protein was induced in small volumes, resulting in no protein signal being detected by Coomassie brilliant blue staining. B, His-TF-GhCASPL1/GhCASPL1* recombinant protein was tested by Western blotting. His-TF-GhCASPL1*, mutation of PA binding motif of GhCASPL1.

**Supplementary Figure 11**


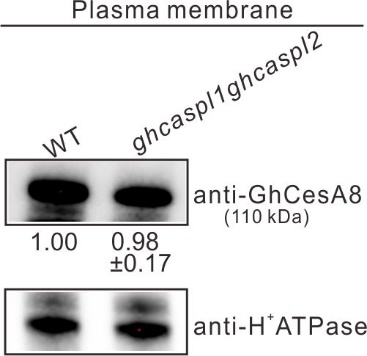


**Supplementary Figure 11 Mutation of *ghcaspl1 ghcaspl2* does not affect the localization of GhCesA8 on the plasma membrane.**

Minute^TM^ Plasma Membrane Protein and Cell Component Isolation Kit (SM-005) was used to isolate the cytoplasmic membrane components of cotton fibers at the secondary wall development stage in both mutant and wild type samples. Subsequently, Western blotting was conducted using the GhCesA8 antibody. H^+^-ATPase as plasma membrane marker.

**Supplementary Figure 12**


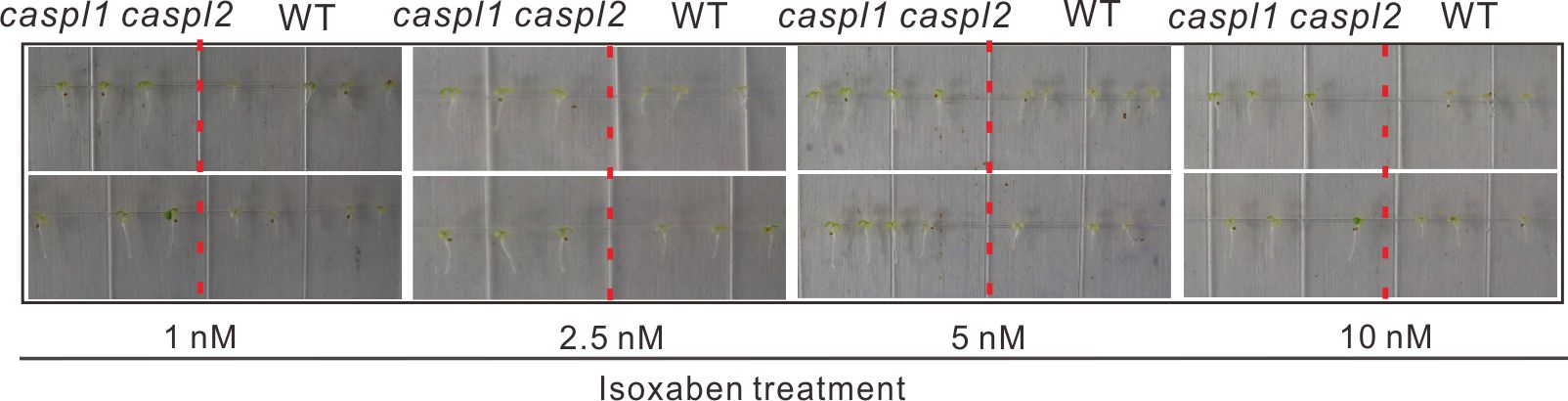


**Supplementary Figure 12 The mutant plants did not show altered sensitivity to the cellulose synthesis inhibitor isoxaben since it produced similar effect on either WT or *caspl1 caspl2* mutant plants.**

Observe the status of wild-type and mutant Arabidopsis after three days of culture in 1/2MS medium supplemented with different concentrations of cellulose inhibitors isoxaben.

| **Table S1 Immunoprecipitation-mass spectrometry (IP-MS) information of 91 shared IP proteins against GhCesA4, GhCesA7, and GhCesA8 antibodies** | | | | | | | | | |
| --- | --- | --- | --- | --- | --- | --- | --- | --- | --- |
| Accession | Annotation | AAs | MW [kDa] | Repeat 1 | | Repeat 2 | | Repeat 3 | |
|  |  |  |  | Coverage | Peptides | Coverage | Peptides | Coverage | Peptides |
| Ghi_D08G10711 | Predicted: tubulin beta-9 chain | 445 | 50.0 | 57.98 | 18 | 49.89 | 15 | 47.87 | 16 |
| Ghi_A11G18716 | Predicted: tubulin beta-5 chain | 447 | 50.3 | 51.90 | 17 | 44.30 | 13 | 45.19 | 16 |
| Ghi_A05G20136 | Glyceraldehyde-3-phosphate dehydrogenase | 287 | 31.5 | 52.96 | 11 | 29.62 | 7 | 44.95 | 8 |
| Ghi_A11G03151 | Predicted: actin-7-like | 377 | 41.7 | 45.89 | 12 | 38.73 | 10 | 30.77 | 9 |
| Ghi_A07G12566 | Hypothetical protein F383_34041 | 373 | 42.8 | 43.43 | 11 | 37.00 | 9 | 13.94 | 4 |
| Ghi_A10G04391 | Phospholipase D alpha 1 | 807 | 91.5 | 22.06 | 13 | 34.94 | 22 | 31.47 | 19 |
| Ghi_D05G01736 | Sucrose synthase 1 | 805 | 92.5 | 31.80 | 20 | 27.20 | 14 | 28.32 | 18 |
| Ghi_A06G10651 | Heat shock protein 70 | 559 | 61.4 | 26.83 | 12 | 32.92 | 14 | 25.58 | 11 |
| Ghi_A12G08606 | Fasciclin-like arabinogalactan protein 4 | 244 | 25.8 | 14.75 | 3 | 38.52 | 5 | 31.97 | 4 |
| Ghi_A12G06241 | Hypothetical protein B456_008G109400 | 148 | 16.5 | 31.76 | 4 | 19.59 | 2 | 31.76 | 4 |
| Ghi_D07G12861 | Predicted: glyceraldehyde-3-phosphate dehydrogenase 2, cytosolic-like | 336 | 36.4 | 32.74 | 10 | 30.36 | 7 | 19.05 | 5 |
| Ghi_D09G09526 | Adenosylhomocysteinase 1 -like protein | 485 | 53.2 | 18.35 | 8 | 44.54 | 12 | 12.99 | 5 |
| Ghi_A01G00866 | Predicted: 26S proteasome non-ATPase regulatory subunit 2 homolog A isoform X1 | 888 | 97.5 | 6.64 | 5 | 18.13 | 8 | 50.79 | 33 |
| Ghi_D06G04306 | Hypothetical protein B456_010G092300 | 818 | 93.9 | 26.04 | 17 | 21.39 | 12 | 24.21 | 16 |
| Ghi_A04G04141 | Hypothetical protein B456_009G407500 | 353 | 38.1 | 20.96 | 7 | 25.50 | 5 | 24.08 | 7 |
| Ghi_D06G04286 | Sucrose synthase isoform C | 796 | 90.3 | 26.88 | 19 | 26.26 | 12 | 14.32 | 11 |
| Ghi_D08G10811 | Predicted: aquaporin PIP1-3-like | 287 | 30.9 | 22.30 | 6 | 28.57 | 5 | 15.68 | 5 |
| Ghi_A05G09161 | Hypothetical protein F383_19968 | 595 | 64.8 | 20.17 | 10 | 23.53 | 11 | 20.84 | 11 |
| Ghi_D04G07986 | Predicted: ATP synthase subunit beta, mitochondrial | 557 | 59.8 | 14.00 | 5 | 32.50 | 10 | 15.08 | 6 |
| Ghi_A08G02811 | Unknown | 263 | 28.1 | 13.69 | 2 | 25.10 | 5 | 21.67 | 4 |
| Ghi_A03G02921 | Hypothetical protein F383_06056 | 164 | 18.2 | 26.83 | 3 | 22.56 | 2 | 10.98 | 1 |
| Ghi_A09G14746 | Predicted: dihydrolipoyl dehydrogenase 2, chloroplastic | 571 | 60.2 | 11.91 | 5 | 16.29 | 5 | 32.05 | 12 |
| Ghi_A12G15586 | T-complex 1 subunit zeta | 535 | 59.0 | 12.90 | 5 | 21.68 | 6 | 25.61 | 10 |
| Ghi_A10G07931 | Serine hydroxymethyltransferase | 471 | 51.9 | 16.77 | 6 | 26.75 | 8 | 16.35 | 6 |
| Ghi_D11G16806 | Predicted: 26S protease regulatory subunit 6A homolog | 423 | 47.5 | 6.62 | 2 | 14.18 | 4 | 38.06 | 12 |
| Ghi_D09G06001 | Arabinogalactan protein 2 | 243 | 25.6 | 19.34 | 4 | 19.34 | 3 | 19.34 | 3 |
| Ghi_D04G05921 | Predicted: fructose-bisphosphate aldolase, cytoplasmic isozyme-like | 403 | 43.9 | 15.38 | 5 | 26.80 | 8 | 14.64 | 5 |
| Ghi_D05G06476 | Dehydrin ERD10 -like protein | 211 | 23.6 | 18.48 | 2 | 18.48 | 2 | 18.48 | 2 |
| Ghi_A09G08746 | Adenosine kinase 2 -like protein | 341 | 37.3 | 14.37 | 4 | 32.26 | 6 | 8.80 | 2 |
| Ghi_A05G05591 | Hypothetical protein F383_20100 | 948 | 105.6 | 24.37 | 20 | 12.45 | 6 | 18.35 | 14 |
| Ghi_A10G04841 | Predicted: T-complex protein 1 subunit gamma | 557 | 60.3 | 25.13 | 11 | 9.69 | 4 | 20.29 | 9 |
| Ghi_A07G12111 | Predicted: putative quinone-oxidoreductase homolog, chloroplastic | 327 | 34.3 | 14.98 | 3 | 31.80 | 6 | 7.34 | 2 |
| Ghi_A09G03481 | Predicted: probable voltage-gated potassium channel subunit beta | 328 | 36.6 | 27.44 | 10 | 18.60 | 3 | 7.62 | 2 |
| Ghi_A11G18086 | Heat shock protein 70 | 816 | 90.5 | 15.20 | 11 | 20.59 | 12 | 15.69 | 11 |
| Ghi_A05G08571 | Predicted: uncharacterized protein LOC105770569 | 165 | 18.7 | 17.58 | 2 | 7.88 | 1 | 25.45 | 3 |
| Ghi_A05G08651 | Hypothetical protein F383_14617 | 939 | 105.8 | 20.34 | 15 | 11.50 | 6 | 18.74 | 13 |
| Ghi_A12G02171 | Predicted: coatomer subunit alpha-1 | 1216 | 136.3 | 25.90 | 28 | 13.57 | 11 | 8.96 | 9 |
| Ghi_A03G04881 | 26S protease regulatory subunit S10B B -like protein | 398 | 44.6 | 8.29 | 3 | 11.06 | 3 | 28.64 | 10 |
| Ghi_A06G03116 | Hypothetical protein B456_010G066100 | 281 | 31.8 | 19.93 | 5 | 16.37 | 4 | 11.03 | 3 |
| Ghi_A11G15631 | Cct8 | 550 | 59.3 | 11.27 | 5 | 18.18 | 6 | 17.64 | 8 |
| Ghi_A03G12751 | Predicted: coatomer subunit alpha-1-like | 1213 | 136.2 | 23.25 | 24 | 12.20 | 11 | 8.08 | 8 |
| Ghi_A09G05291 | Coatomer subunit beta | 1134 | 128.2 | 17.11 | 15 | 9.52 | 6 | 15.52 | 13 |
| Ghi_D10G02376 | hypothetical protein B456_011G053900 | 998 | 109.9 | 13.43 | 12 | 15.93 | 12 | 12.22 | 11 |
| Ghi_A05G13636 | Cotton annexin 3 | 316 | 35.9 | 6.96 | 2 | 27.53 | 7 | 6.96 | 2 |
| Ghi_A04G07486 | Predicted: 60S ribosomal protein L3-2 isoform X1 | 389 | 44.3 | 4.11 | 2 | 10.03 | 4 | 26.99 | 12 |
| Ghi_A11G16091 | Predicted: 40S ribosomal protein S21-like | 82 | 9.3 | 13.41 | 1 | 13.41 | 1 | 13.41 | 1 |
| Ghi_A07G06681 | Arabinogalactan protein 4 | 239 | 25.4 | 10.88 | 2 | 17.99 | 2 | 10.88 | 2 |
| Ghi_A01G09576 | Predicted: probable 26S proteasome non-ATPase regulatory subunit 3 | 566 | 64.9 | 7.07 | 4 | 4.95 | 2 | 26.86 | 15 |
| Ghi_D08G02686 | Predicted: fasciclin-like arabinogalactan protein 7 | 261 | 27.9 | 3.83 | 1 | 21.84 | 4 | 13.03 | 3 |
| Ghi_A12G02361 | Hypothetical protein F383_17048 | 479 | 53.9 | 18.58 | 7 | 10.44 | 4 | 8.77 | 3 |
| Ghi_A06G04011 | Predicted: nucleosome assembly protein 1;2 | 364 | 41.4 | 18.96 | 6 | 4.95 | 1 | 13.74 | 3 |
| Ghi_D11G09191 | Hypothetical protein B456_007G188400 | 506 | 55.7 | 14.23 | 6 | 17.39 | 7 | 4.55 | 2 |
| Ghi_A02G09721 | 60S ribosomal L8-3 -like protein | 260 | 28.1 | 7.31 | 1 | 7.31 | 1 | 21.54 | 4 |
| Ghi_A11G18181 | fasciclin-like arabinogalactan protein 6 | 241 | 25.7 | 3.73 | 1 | 14.52 | 2 | 17.84 | 3 |
| Ghi_A01G02301 | Phosphoenolpyruvate carboxylase, housekeeping isozyme | 969 | 110.6 | 10.73 | 8 | 12.59 | 8 | 12.07 | 10 |
| Ghi_A07G12321 | 26S proteasome regulatory subunit 4 A -like protein | 446 | 49.6 | 5.61 | 2 | 7.40 | 2 | 22.20 | 7 |
| Ghi_A03G01516 | cytosolic pyruvate kinase 1 | 506 | 55.0 | 18.97 | 8 | 12.45 | 4 | 2.77 | 1 |
| Ghi_A10G13446 | UDP-glucuronic acid decarboxylase 1 | 368 | 41.9 | 10.87 | 4 | 20.65 | 3 | 1.90 | 1 |
| Ghi_A09G12871 | Predicted: chaperonin CPN60-2, mitochondrial isoform X1 | 575 | 61.4 | 18.61 | 9 | 12.00 | 4 | 1.91 | 1 |
| Ghi_A09G13016 | Predicted: 26S proteasome non-ATPase regulatory subunit 1 homolog A isoform X1 | 1028 | 111.9 | 2.43 | 2 | 11.09 | 8 | 18.68 | 13 |
| Ghi_A11G09446 | Isocitrate dehydrogenase [NADP] | 411 | 46.0 | 10.71 | 4 | 18.98 | 7 | 2.43 | 1 |
| Ghi_A05G09436 | Regulatory particle triple-A 1A | 426 | 47.7 | 3.05 | 1 | 10.09 | 3 | 18.78 | 7 |
| Ghi_A05G01406 | Predicted: ras-related protein RABE1c | 271 | 30.5 | 17.71 | 4 | 5.17 | 1 | 8.12 | 2 |
| Ghi_D03G04381 | Predicted: villin-2 | 1153 | 126.5 | 15.78 | 14 | 13.01 | 11 | 1.39 | 2 |
| Ghi_A11G02996 | Annexin D2 -like protein | 316 | 35.7 | 3.16 | 1 | 17.41 | 4 | 9.18 | 2 |
| Ghi_A08G12021 | Elongation factor 1-delta | 290 | 32.2 | 9.66 | 4 | 14.14 | 3 | 5.86 | 2 |
| Ghi_D10G06286 | Predicted: T-complex protein 1 subunit alpha | 545 | 59.1 | 12.66 | 6 | 4.04 | 2 | 12.84 | 6 |
| Ghi_D05G12946 | Predicted: elongation factor 2-like | 874 | 97.3 | 9.84 | 8 | 17.62 | 10 | 2.06 | 2 |
| Ghi_D11G00501 | Predicted: tripeptidyl-peptidase 2-like | 1346 | 148.9 | 8.77 | 11 | 5.79 | 5 | 13.37 | 16 |
| Ghi_A12G03576 | Predicted: succinate dehydrogenase [ubiquinone] flavoprotein subunit 1 | 633 | 69.8 | 4.27 | 2 | 4.58 | 2 | 18.80 | 7 |
| Ghi_A13G07041 | 5-methyltetrahydropteroyltriglutamate--homocysteine methyltransferase | 770 | 85.3 | 7.01 | 5 | 13.38 | 7 | 6.88 | 5 |
| Ghi_A02G01601 | Predicted: CASP-like protein 1D1 | 204 | 21.7 | 4.41 | 1 | 11.27 | 2 | 11.27 | 2 |
| Ghi_D01G04351 | Predicted: catalase isozyme 1 | 490 | 56.5 | 10.20 | 4 | 10.82 | 3 | 5.92 | 3 |
| Ghi_D10G00496 | Predicted: pyrophosphate--fructose 6-phosphate 1-phosphotransferase subunit beta | 566 | 61.8 | 7.42 | 4 | 9.19 | 3 | 8.30 | 4 |
| Ghi_A06G11106 | Polyphenol oxidase-7 | 616 | 70.3 | 9.09 | 4 | 2.76 | 1 | 12.99 | 7 |
| Ghi_A01G08736 | Predicted: T-complex protein 1 subunit epsilon | 535 | 59.2 | 9.91 | 5 | 10.47 | 2 | 4.11 | 2 |
| Ghi_D11G00961 | Predicted: hsp70-Hsp90 organizing protein 3 | 582 | 65.4 | 11.86 | 5 | 1.55 | 1 | 7.90 | 4 |
| Ghi_A09G10026 | Predicted: V-type proton ATPase subunit B 1 | 522 | 58.1 | 3.83 | 1 | 2.87 | 1 | 9.39 | 3 |
| Ghi_D09G04781 | Predicted: phospholipid:diacylglycerol acyltransferase 1-like | 1135 | 127.0 | 5.81 | 6 | 7.22 | 5 | 2.47 | 3 |
| Ghi_A11G16556 | ATP-citrate synthase alpha chain 3 -like protein | 423 | 46.6 | 7.33 | 3 | 5.44 | 2 | 2.60 | 1 |
| Ghi_A08G14801 | Predicted: peroxisomal fatty acid beta-oxidation multifunctional protein AIM1-like isoform X1 | 724 | 78.3 | 5.80 | 4 | 7.60 | 3 | 1.66 | 1 |
| Ghi_A09G08016 | Predicted: aspartic proteinase nepenthesin-2 | 468 | 51.1 | 3.21 | 1 | 2.35 | 1 | 8.76 | 4 |
| Ghi_A11G08021 | Auxin-induced in root cultures 12 -like protein | 398 | 43.3 | 2.76 | 1 | 5.28 | 2 | 5.03 | 2 |
| Ghi_A07G04161 | Predicted: putative endo-1,3(4)-beta-glucanase 2 | 732 | 81.7 | 5.19 | 3 | 5.05 | 3 | 1.50 | 1 |
| Ghi_A05G24006 | Hypothetical protein F383_09865 | 453 | 49.9 | 2.65 | 1 | 6.40 | 2 | 2.65 | 1 |
| Ghi_A06G02521 | Predicted: molybdate transporter 1-like | 451 | 48.2 | 4.66 | 2 | 2.00 | 1 | 2.66 | 1 |
| Ghi_A09G07791 | Predicted: eukaryotic translation initiation factor 3 subunit C-like isoform X1 | 909 | 102.5 | 2.20 | 2 | 2.86 | 2 | 3.63 | 3 |
| Ghi_A02G07571 | Hypothetical protein B456_003G041200 | 829 | 94.1 | 2.90 | 2 | 2.05 | 1 | 2.90 | 2 |
| Ghi_A11G02291 | Nuclease domain-containing 1 | 997 | 108.5 | 2.11 | 2 | 4.61 | 2 | 1.10 | 1 |
| Ghi_D12G11021 | Predicted: phosphoenolpyruvate carboxylase 4 | 1055 | 118.4 | 1.99 | 2 | 1.14 | 1 | 2.09 | 2 |
| Ghi_D10G11206 | Predicted: exportin-7 isoform X1 | 1659 | 183.8 | 0.78 | 1 | 2.29 | 2 | 1.51 | 2 |

**Table S2 The expression patterns of 91 shared *GhCesAs*-IP proteins across different tissues of cotton**

| Gene | root | stem | leaf | petal | 5DPA_Ovule | 10DPA_Ovule | 15DPA_Ovule | 20DPA_Ovule | 25DPA_Ovule | 10DPA_Fiber | 15DPA_Fiber | 20DPA_Fiber | 25DPA_Fiber |
| --- | --- | --- | --- | --- | --- | --- | --- | --- | --- | --- | --- | --- | --- |
| *GhFLA2* | 2 | 0 | 1 | 4 | 2 | 33 | 141 | 84 | 363 | 9 | 2.49e+3 | 1.31e+4 | 2.42e+4 |
| *GhFLA7* | 2 | 1 | 1 | 2 | 1 | 4 | 8 | 75 | 426 | 3 | 135 | 5.62e+3 | 1.07e+4 |
| *GhFLA1* | 96 | 26 | 26 | 72 | 70 | 277 | 375 | 90 | 167 | 662 | 1.81e+3 | 2.56e+3 | 8.71e+3 |
| *GhAP2* | 88 | 325 | 7 | 12 | 1.13e+3 | 361 | 529 | 73 | 26 | 1.47e+3 | 5.24e+3 | 6.91e+3 | 7.34e+3 |
| *GhCASPL1* | 13 | 8 | 8 | 3 | 1 | 12 | 20 | 15 | 55 | 12 | 497 | 3.44e+3 | 6.64e+3 |
| GhAP4 | 24 | 113 | 0 | 2 | 0 | 3 | 4 | 1 | 6 | 3 | 74 | 5.45e+3 | 3.26e+3 |
| *GhTubulin* | 127 | 375 | 60 | 10 | 176 | 239 | 358 | 38 | 29 | 373 | 995 | 1.74e+3 | 1.09e+3 |
| *GhDehydrin* | 632 | 539 | 157 | 1.22e+3 | 230 | 771 | 340 | 125 | 77 | 371 | 409 | 1.49e+3 | 970 |
| *GhFLA6* | 50 | 121 | 2 | 1 | 0 | 11 | 27 | 0 | 2 | 4 | 352 | 1.47e+3 | 253 |
| *GhX1* | 404 | 408 | 97 | 359 | 507 | 599 | 547 | 205 | 124 | 498 | 528 | 966 | 562 |
| Ghi_D08G10811 | 308 | 475 | 317 | 145 | 1.28e+3 | 865 | 681 | 532 | 239 | 4.12e+3 | 1.80e+3 | 1.13e+3 | 213 |
| Ghi_A04G04141 | 392 | 369 | 85 | 134 | 454 | 502 | 1.04e+3 | 615 | 415 | 265 | 383 | 753 | 514 |
| Ghi_D08G10711 | 39 | 54 | 10 | 13 | 529 | 302 | 373 | 58 | 24 | 1.45e+3 | 2.42e+3 | 780 | 277 |
| Ghi_A05G20136 | 631 | 708 | 921 | 267 | 1.35e+3 | 560 | 1.12e+3 | 533 | 834 | 1.13e+3 | 925 | 673 | 318 |
| Ghi_D06G04286 | 23 | 82 | 1 | 2 | 2 | 30 | 60 | 2 | 2 | 86 | 328 | 609 | 232 |
| Ghi_A05G13636 | 156 | 276 | 98 | 274 | 597 | 281 | 434 | 83 | 121 | 817 | 656 | 422 | 307 |
| Ghi_A11G18086 | 513 | 439 | 35 | 18 | 457 | 367 | 645 | 115 | 352 | 490 | 505 | 170 | 454 |
| Ghi_A11G08021 | 39 | 67 | 2 | 12 | 61 | 94 | 195 | 12 | 3 | 67 | 297 | 503 | 85 |
| Ghi_D05G01736 | 789 | 820 | 27 | 66 | 938 | 169 | 295 | 203 | 17 | 196 | 108 | 428 | 99 |
| Ghi_D04G05921 | 259 | 215 | 64 | 114 | 427 | 262 | 318 | 219 | 92 | 149 | 216 | 344 | 176 |
| Ghi_A06G10651 | 345 | 328 | 88 | 149 | 171 | 205 | 167 | 211 | 77 | 226 | 215 | 186 | 293 |
| Ghi_A11G03151 | 175 | 191 | 42 | 33 | 332 | 250 | 227 | 167 | 92 | 313 | 286 | 225 | 227 |
| Ghi_A12G06241 | 262 | 243 | 272 | 74 | 896 | 740 | 467 | 418 | 112 | 1.21e+3 | 620 | 401 | 33 |
| Ghi_D01G04351 | 103 | 168 | 73 | 664 | 100 | 123 | 163 | 79 | 56 | 131 | 204 | 223 | 167 |
| Ghi_A10G07931 | 214 | 430 | 55 | 34 | 382 | 269 | 508 | 139 | 80 | 159 | 180 | 135 | 242 |
| Ghi_D09G09526 | 252 | 456 | 91 | 27 | 909 | 260 | 440 | 147 | 132 | 518 | 274 | 218 | 155 |
| Ghi_A09G08746 | 110 | 190 | 129 | 36 | 418 | 129 | 201 | 84 | 77 | 285 | 260 | 201 | 139 |
| Ghi_D04G07986 | 176 | 146 | 55 | 119 | 274 | 287 | 177 | 195 | 41 | 129 | 138 | 213 | 127 |
| Ghi_D06G04306 | 98 | 49 | 2 | 16 | 158 | 200 | 75 | 33 | 3 | 44 | 51 | 201 | 114 |
| Ghi_A10G13446 | 90 | 196 | 9 | 75 | 43 | 86 | 402 | 17 | 49 | 24 | 110 | 108 | 203 |
| Ghi_A09G03481 | 131 | 118 | 68 | 53 | 246 | 140 | 145 | 71 | 53 | 199 | 175 | 150 | 109 |
| Ghi_A02G09721 | 306 | 274 | 120 | 39 | 273 | 353 | 317 | 237 | 341 | 155 | 190 | 92 | 143 |
| Ghi_A10G04391 | 174 | 152 | 33 | 244 | 228 | 254 | 211 | 103 | 22 | 231 | 176 | 146 | 79 |
| Ghi_A03G01516 | 47 | 30 | 8 | 36 | 52 | 41 | 28 | 14 | 8 | 64 | 58 | 139 | 76 |
| Ghi_D11G09191 | 104 | 138 | 54 | 58 | 230 | 112 | 248 | 66 | 43 | 127 | 154 | 122 | 88 |
| Ghi_A09G08016 | 22 | 31 | 22 | 41 | 641 | 176 | 198 | 54 | 17 | 667 | 837 | 161 | 45 |
| Ghi_A11G09446 | 171 | 118 | 40 | 280 | 318 | 180 | 200 | 83 | 57 | 179 | 144 | 119 | 85 |
| Ghi_A11G16091 | 256 | 171 | 221 | 70 | 234 | 124 | 160 | 61 | 400 | 130 | 163 | 64 | 134 |
| Ghi_A06G03116 | 88 | 96 | 39 | 78 | 196 | 116 | 105 | 73 | 59 | 265 | 186 | 128 | 51 |
| Ghi_A11G16556 | 49 | 51 | 26 | 62 | 174 | 95 | 125 | 38 | 11 | 97 | 97 | 129 | 26 |
| Ghi_A12G02361 | 184 | 175 | 75 | 32 | 160 | 168 | 229 | 107 | 199 | 120 | 144 | 81 | 71 |
| Ghi_D11G00961 | 41 | 16 | 6 | 7 | 70 | 32 | 25 | 22 | 9 | 108 | 53 | 80 | 49 |
| Ghi_D05G12946 | 156 | 136 | 58 | 64 | 145 | 133 | 108 | 124 | 33 | 60 | 66 | 76 | 52 |
| Ghi_A05G24006 | 2 | 5 | 15 | 1 | 136 | 8 | 5 | 22 | 7 | 41 | 16 | 106 | 19 |
| Ghi_D03G04381 | 26 | 24 | 5 | 20 | 42 | 24 | 22 | 12 | 2 | 41 | 42 | 68 | 53 |
| Ghi_A04G07486 | 22 | 15 | 26 | 8 | 82 | 56 | 54 | 42 | 101 | 73 | 63 | 51 | 58 |
| Ghi_A08G14801 | 40 | 33 | 10 | 150 | 33 | 50 | 51 | 29 | 14 | 32 | 34 | 51 | 55 |
| Ghi_A11G02996 | 43 | 40 | 8 | 36 | 90 | 85 | 107 | 16 | 17 | 66 | 82 | 49 | 54 |
| Ghi_A07G12321 | 53 | 42 | 16 | 37 | 60 | 50 | 57 | 40 | 15 | 34 | 48 | 64 | 40 |
| Ghi_A06G04011 | 68 | 65 | 26 | 30 | 86 | 59 | 44 | 62 | 27 | 54 | 55 | 58 | 42 |
| Ghi_A05G05591 | 32 | 29 | 7 | 11 | 49 | 35 | 45 | 28 | 11 | 26 | 42 | 53 | 45 |
| Ghi_A12G03576 | 51 | 62 | 19 | 118 | 53 | 68 | 43 | 37 | 9 | 44 | 47 | 64 | 32 |
| Ghi_A01G00866 | 54 | 46 | 12 | 26 | 53 | 58 | 44 | 39 | 11 | 35 | 39 | 49 | 44 |
| Ghi_D11G16806 | 52 | 49 | 22 | 44 | 61 | 31 | 47 | 20 | 21 | 28 | 53 | 39 | 54 |
| Ghi_D09G04781 | 60 | 59 | 18 | 36 | 62 | 48 | 32 | 30 | 11 | 40 | 41 | 53 | 35 |
| Ghi_A01G08736 | 55 | 49 | 22 | 11 | 94 | 64 | 49 | 60 | 20 | 63 | 57 | 51 | 29 |
| Ghi_A10G04841 | 68 | 54 | 23 | 13 | 113 | 47 | 55 | 45 | 34 | 49 | 53 | 37 | 44 |
| Ghi_A03G04881 | 54 | 50 | 44 | 36 | 69 | 53 | 63 | 34 | 70 | 35 | 39 | 40 | 34 |
| Ghi_A08G12021 | 67 | 74 | 116 | 40 | 116 | 50 | 52 | 63 | 80 | 88 | 72 | 48 | 26 |
| Ghi_D07G12861 | 39 | 37 | 42 | 21 | 72 | 53 | 60 | 63 | 95 | 71 | 67 | 36 | 37 |
| Ghi_A09G07791 | 34 | 29 | 10 | 25 | 55 | 48 | 28 | 39 | 7 | 25 | 29 | 42 | 29 |
| Ghi_A07G04161 | 31 | 23 | 2 | 122 | 70 | 21 | 23 | 9 | 2 | 60 | 87 | 51 | 17 |
| Ghi_A05G09436 | 54 | 44 | 22 | 43 | 61 | 46 | 72 | 30 | 55 | 42 | 48 | 27 | 33 |
| Ghi_A09G14746 | 21 | 14 | 7 | 4 | 43 | 41 | 36 | 83 | 21 | 31 | 27 | 34 | 26 |
| Ghi_A05G09161 | 37 | 34 | 19 | 46 | 35 | 23 | 23 | 14 | 6 | 15 | 27 | 32 | 26 |
| Ghi_A01G09576 | 58 | 52 | 15 | 30 | 55 | 33 | 37 | 33 | 16 | 20 | 34 | 26 | 31 |
| Ghi_A07G12111 | 125 | 51 | 6 | 148 | 2 | 45 | 28 | 4 | 3 | 81 | 96 | 53 | 3 |
| Ghi_A11G02291 | 39 | 34 | 9 | 26 | 30 | 53 | 53 | 112 | 23 | 18 | 24 | 39 | 16 |
| Ghi_A09G10026 | 25 | 29 | 9 | 24 | 32 | 38 | 28 | 20 | 11 | 21 | 28 | 31 | 24 |
| Ghi_A05G01406 | 60 | 51 | 25 | 76 | 63 | 68 | 74 | 30 | 24 | 37 | 52 | 28 | 27 |
| Ghi_D10G06286 | 64 | 52 | 23 | 13 | 76 | 52 | 49 | 46 | 43 | 35 | 36 | 30 | 21 |
| Ghi_A02G07571 | 41 | 27 | 42 | 10 | 53 | 30 | 29 | 30 | 33 | 39 | 37 | 29 | 21 |
| Ghi_A03G02921 | 62 | 69 | 39 | 147 | 30 | 36 | 24 | 12 | 0 | 11 | 13 | 50 | 0 |
| Ghi_A12G15586 | 45 | 40 | 19 | 7 | 69 | 37 | 26 | 39 | 14 | 33 | 25 | 24 | 23 |
| Ghi_A12G02171 | 20 | 17 | 3 | 9 | 27 | 30 | 24 | 21 | 2 | 25 | 29 | 29 | 15 |
| Ghi_A09G05291 | 16 | 15 | 4 | 8 | 22 | 24 | 22 | 22 | 7 | 20 | 20 | 27 | 16 |
| Ghi_A11G15631 | 51 | 45 | 22 | 9 | 81 | 52 | 32 | 57 | 17 | 54 | 29 | 25 | 14 |
| Ghi_D10G00496 | 25 | 28 | 6 | 5 | 62 | 41 | 68 | 18 | 7 | 16 | 9 | 22 | 16 |
| Ghi_D10G11206 | 25 | 38 | 39 | 13 | 43 | 37 | 28 | 43 | 17 | 27 | 16 | 18 | 19 |
| Ghi_A05G08651 | 17 | 16 | 6 | 7 | 17 | 18 | 19 | 18 | 10 | 11 | 15 | 20 | 12 |
| Ghi_A09G13016 | 33 | 25 | 5 | 20 | 33 | 33 | 27 | 27 | 6 | 12 | 15 | 15 | 15 |
| Ghi_A05G08571 | 61 | 66 | 64 | 14 | 109 | 14 | 18 | 22 | 45 | 22 | 10 | 8 | 17 |
| Ghi_A09G12871 | 25 | 20 | 15 | 12 | 43 | 20 | 17 | 22 | 11 | 34 | 15 | 13 | 12 |
| Ghi_A01G02301 | 121 | 168 | 45 | 12 | 53 | 15 | 9 | 7 | 2 | 15 | 11 | 9 | 14 |
| Ghi_D10G02376 | 10 | 10 | 6 | 26 | 65 | 30 | 13 | 8 | 1 | 59 | 41 | 6 | 16 |
| Ghi_D12G11021 | 16 | 16 | 1 | 6 | 33 | 45 | 25 | 20 | 2 | 17 | 13 | 14 | 7 |
| Ghi_A03G12751 | 12 | 12 | 3 | 7 | 10 | 15 | 15 | 12 | 3 | 7 | 11 | 13 | 8 |
| Ghi_A13G07041 | 21 | 18 | 5 | 3 | 27 | 13 | 9 | 11 | 6 | 5 | 8 | 9 | 12 |
| Ghi_D11G00501 | 28 | 22 | 4 | 14 | 35 | 46 | 28 | 47 | 7 | 27 | 20 | 9 | 6 |
| Ghi_A06G02521 | 2 | 4 | 1 | 1 | 1 | 2 | 1 | 1 | 1 | 0 | 1 | 5 | 3 |
| Ghi_A06G11106 | 0 | 0 | 15 | 0 | 158 | 0 | 0 | 0 | 0 | 0 | 0 | 0 | 0 |

**Table S3 Primers used in this study**

| qRT-PCR | Forward primer/Reverse primer |
| --- | --- |
| UBQ7 | AGAGGTCGAGTCTTCGGACA/TGCTTGATCTTCTTGGGCTT |
| GhCesA8 | GCATTGCCCTCTATGGTATGGCTTT/AAACTGCTCGTTACGCCATAAGT |
| GhCASPL1 | TAAAGGGCAACACTCACGTCG/CAGAAGGATGGCAGCAAACAAT |
| GhFLA2 | CCGTCACCCTCACCAGCACCGG/AGGCGGACGGTACCAGACTCAT |
| GhFLA1 | TGAGGATTTCGGCCTCAACGTA/ACCCCATATTCCTTCCGGTTGAT |
| GhFLA7 | CCCTCACCGGCACCGACAC/CGGACGGTACCAGAATCGT |
| GhAP2 | CCGGCACTGGCCCCGGCAC/GCCCGATTTAAGGCTGGAG |
| Luciferase assays | Forward primer/Reverse primer |
| GhCesA8 to N/C-LUC | gtcccggggcggtaccATGATGGAATCTGGGGTTCCTGT/gagaagagccgggcccACAATCAATGGAAATGCAGCTCTGT |
| GhCASPL1 to N/C-LUC | gtcccggggcggtaccATGGCATCCACTGATAAAACTGCTG/gagaagagccgggcccGTCGCGGATCTTTTTGTAAAGTGT |
| CesA4 to N/C-LUC | gtcccggggcggtaccATGGAACCAAACACCATGGCCAGCTTCG/gagaagagccgggcccACAGTCGACGCCACATTGCT |
| CASPL1 to N/C-LUC | gtcccggggcggtaccATGGGTTATGAAACCAAATCGACTTTGGAC/gagaagagccgggcccACGGATTTTCTTGTAGAGGGAGAGG |
| CASPL2 to N/C-LUC | gtcccggggcggtaccATGGCGTCTACTGAGAACCCTGACCCTG/gagaagagccgggcccTGTTCTCTTGGAGAGTGCTGACCAG |
| TWD40 to N/C-LUC | gtcccggggcggtaccATGTTGCGGGCGAGAGCATTTCGGC/gagaagagccgggcccGCCATTCAAGAAATCAAAATC |
| The DUAL membrane yeast two-hybrid assay | Forward primer/Reverse primer |
| GhCesA8 to ppR3-N | atgttccagattacgctggatccATGATGGAATCTGGGGTTCCTGT/gtatcgataagcttgatatcgaattcACAATCAATGGAAATGCAGCTCTGT |
| GhCesA7 to ppR3-N | atgttccagattacgctggatccATGGAAGCCAGCGCCGGACTCGTCGC/gtatcgataagcttgatatcgaattcACAGTTAATGCCACAC |
| GhCesA4 to ppR3-N | atgttccagattacgctggatccATGGCTTCAACCACCATGGCCGCTGGC/gtatcgataagcttgatatcgaattcGCACTCCACGCCACATTG |
| GhCASPL1 to pBT3-SUC | aatatctgcaatggccattacggccATGGCTTCTACTGACAAGACTGC/tgcagatggccgaggcggccccGTCTCTGATCTTCTTGTACAAAGTGAAAG |
| Identification of cotton mutants | Forward primer/Reverse primer |
| GhCASPL1_At_IDEN | CGACAATCACGGGGGAAGTAGGG/ATATAGTCCTGAATTTAGGCC |
| GhCASPL1_Dt_IDEN | GTGCGACAATCATGCGGGAAGTAAGG/GAAAATTATAGTCTTGGTTTAGGCC |
| GhCASPL2_At_IDEN | GAATGGCGTGCGGGGAATTCCAACC/GGGTTTGTTAACTCAACTCGATTTGACTC |
| GhCASPL2_Dt_IDEN | GAATGGCGTGCGGGGAATTCCAACC/CGAATATAACCAAACCCGAGTATAAGGTA |
| Identification of *Arabidopsis* mutants | Forward primer/Reverse primer |
| CASPL1_IDEN | CGACTTTGGACACCGAGAGATCGA/GGATTTTCTTGTAGAGGGAGAGG |
| CASPL2_IDEN | ACAGTCCAAACTTAACCAC/CACAGACAAACACGTGTGAAC |
| Identification of *GhCASPL1* heterologous expression | Forward primer/Reverse primer |
| Check-F/R | GCCCGACAACCACTACCTGAGC/GTCGCGGATCTTTTTGTAAAG |
| ArUBQ5 | GGTGCTAAGAAGAGGAAGAAGA/CCGCTACGTTGCCATTGC |
| Subcellular localization | Forward primer/Reverse primer |
| GhCASPL1/GhCASPL1*-YFP | tcgtacgcgtcccgggaaATGGCATCCACTGATAAAAC/aacgaaagctctgcagtcaGTCTGCGATTGCAGCG |
| GhCesA8-mCherry | TGCAGGGGCCCGGGGTCGACATGATGGAATCTGGGGTTCCTGT/CATGGTACCGGATCCACTAGTACAATCAATGGAAATGCAGCTCTGT |

| **Table S4 Differentially expressed genes of wild type and g*hcaspl1 ghcaspl2* mutants in the cotton fiber during SCW development** | | | | | | | | | | | | | | |
| --- | --- | --- | --- | --- | --- | --- | --- | --- | --- | --- | --- | --- | --- | --- |
| Gene ID | Annotation | sampleA | sampleB | logFC | logCPM | PValue | FDR | sampleA | sampleB | logFC | logCPM | PValue | FDR | Putative transmembrane frequency |
| Ghi_D01G10781 | PREDICTED: secoisolariciresinol dehydrogenase-like | Mu1 | WT | 3.760 | 2.128 | 0.000 | 0.000 | Mu2 | WT | 3.47 | 1.925 | 0.000 | 0.001 | 0 |
| Ghi_D11G04361 | PREDICTED: uncharacterized protein LOC105800915 | Mu1 | WT | 3.226 | 2.819 | 0.000 | 0.000 | Mu2 | WT | 3.91 | 3.509 | 0.000 | 0.000 | 0 |
| Ghi_D01G11296 | PREDICTED: phenylalanine ammonia-lyase-like | Mu1 | WT | 2.661 | 2.962 | 0.000 | 0.000 | Mu2 | WT | 3.78 | 4.034 | 0.000 | 0.000 | 0 |
| Ghi_A01G12166 | PAL | Mu1 | WT | 2.523 | 2.925 | 0.000 | 0.000 | Mu2 | WT | 3.77 | 4.097 | 0.000 | 0.000 | 0 |
| Ghi_A05G00596 | Bile acid-inducible operon CD | Mu1 | WT | 2.699 | 1.550 | 0.000 | 0.001 | Mu2 | WT | 3.26 | 2.104 | 0.002 | 0.036 | 0 |
| Ghi_D07G00721 | GHMYB38 | Mu1 | WT | 2.625 | 1.453 | 0.000 | 0.000 | Mu2 | WT | 3.26 | 2.068 | 0.000 | 0.003 | 0 |
| Ghi_D12G00956 | PREDICTED: uncharacterized protein LOC105762766 isoform X1 | Mu1 | WT | 2.594 | 1.286 | 0.000 | 0.000 | Mu2 | WT | 2.93 | 1.631 | 0.000 | 0.012 | 0 |
| Ghi_A11G03876 | NAC domain protein 12 | Mu1 | WT | 2.060 | 4.435 | 0.001 | 0.008 | Mu2 | WT | 2.83 | 5.143 | 0.000 | 0.004 | 0 |
| Ghi_A09G10046 | Peroxidase | Mu1 | WT | 2.964 | 4.445 | 0.000 | 0.000 | Mu2 | WT | 2.69 | 4.267 | 0.000 | 0.008 | 0 |
| Ghi_D13G04131 | PREDICTED: abrin-b-like | Mu1 | WT | 2.416 | 4.365 | 0.000 | 0.000 | Mu2 | WT | 2.50 | 4.499 | 0.000 | 0.001 | 0 |
| Ghi_A11G14596 | NAC protein 8 | Mu1 | WT | 2.211 | 4.430 | 0.000 | 0.005 | Mu2 | WT | 2.46 | 4.699 | 0.001 | 0.026 | 0 |
| Ghi_D10G05256 | PREDICTED: ethylene-responsive transcription factor TINY-like | Mu1 | WT | 2.888 | 2.125 | 0.000 | 0.000 | Mu2 | WT | 2.40 | 1.776 | 0.001 | 0.029 | 0 |
| Ghi_A09G08186 | PREDICTED: zinc finger A20 and AN1 domain-containing stress-associated protein 6-like isoform X4 | Mu1 | WT | 2.370 | 2.635 | 0.000 | 0.000 | Mu2 | WT | 2.32 | 2.653 | 0.000 | 0.000 | 0 |
| Ghi_A09G10636 | hypothetical protein B456_006G204200 | Mu1 | WT | 2.016 | 1.503 | 0.000 | 0.000 | Mu2 | WT | 2.23 | 1.734 | 0.000 | 0.000 | 0 |
| Ghi_A03G04821 | hypothetical protein B456_005G108200 | Mu1 | WT | -2.897 | 3.074 | 0.000 | 0.000 | Mu2 | WT | -2.26 | 3.232 | 0.000 | 0.000 | 0 |
| Ghi_A10G08081 | PREDICTED: tetrahydrocannabinolic acid synthase-like | Mu1 | WT | -2.167 | 7.924 | 0.000 | 0.000 | Mu2 | WT | -2.35 | 7.961 | 0.000 | 0.000 | 0 |
| Ghi_D11G03651 | putative xyloglucan endotransglucosylase/hydrolase B | Mu1 | WT | -2.112 | 4.341 | 0.000 | 0.000 | Mu2 | WT | -2.35 | 4.365 | 0.000 | 0.000 | 1 |
| Ghi_D01G00816 | PREDICTED: leucine-rich repeat extensin-like protein 4 | Mu1 | WT | -2.057 | 7.658 | 0.000 | 0.000 | Mu2 | WT | -2.39 | 7.666 | 0.000 | 0.000 | 0 |
| Ghi_D02G01541 | PREDICTED: non-specific lipid-transfer protein-like protein | Mu1 | WT | -2.829 | 7.222 | 0.000 | 0.000 | Mu2 | WT | -2.95 | 7.274 | 0.000 | 0.009 | 0 |
| Ghi_A07G09756 | hypothetical protein B456_N003400 | Mu1 | WT | -2.343 | 1.978 | 0.000 | 0.000 | Mu2 | WT | -3.16 | 1.939 | 0.000 | 0.000 | 0 |
| Ghi_D09G04371 | PREDICTED: stem-specific protein TSJT1-like | Mu1 | WT | -2.101 | 4.973 | 0.000 | 0.000 | Mu2 | WT | -3.18 | 4.888 | 0.000 | 0.000 | 0 |
| Ghi_D03G08156 | PREDICTED: probable aquaporin PIP2-2 | Mu1 | WT | -2.563 | 5.105 | 0.000 | 0.000 | Mu2 | WT | -3.20 | 5.096 | 0.000 | 0.003 | 6 |
| Ghi_D12G00516 | PREDICTED: desiccation-related protein PCC13-62-like | Mu1 | WT | -3.027 | 7.489 | 0.000 | 0.000 | Mu2 | WT | -3.38 | 7.523 | 0.000 | 0.000 | 0 |
| Ghi_D12G08691 | PREDICTED: ethylene response sensor 1-like | Mu1 | WT | -3.098 | 6.183 | 0.000 | 0.000 | Mu2 | WT | -3.44 | 6.218 | 0.000 | 0.000 | 3 |
| Ghi_A12G00241 | PREDICTED: non-specific lipid-transfer protein-like protein At2g13820 isoform X1 | Mu1 | WT | -3.804 | 2.744 | 0.000 | 0.000 | Mu2 | WT | -3.50 | 2.836 | 0.000 | 0.000 | 0 |
| Ghi_D11G04471 | PREDICTED: tubulin alpha-3 chain-like | Mu1 | WT | -6.519 | 6.962 | 0.000 | 0.000 | Mu2 | WT | -6.45 | 7.029 | 0.000 | 0.000 | 0 |
| Note: Predict protein transmembrane domains by DeepTMHMM - Predictions Website; The table shows the differentially expressed genes with logFC > 2, FPKM > 20 and FDR < 0.05. | | | | | | | | | | | | | | |
